# Efficient launching of shear phonons in photostrictive halide perovskites

**DOI:** 10.1126/sciadv.adw9172

**Published:** 2025-11-05

**Authors:** Dmytro O. Horiachyi, Mikhail O. Nestoklon, Ilya A. Akimov, Artur V. Trifonov, Nikita V. Siverin, Nataliia E. Kopteva, Alexander N. Kosarev, Dmitri R. Yakovlev, Vitalyi E. Gusev, Melina Fries, Olga Trukhina, Vladimir Dyakonov, Manfred Bayer

**Affiliations:** ^1^ Experimentelle Physik 2, Technische Universität Dortmund, 44221 Dortmund, Germany.; ^2^ Institut für Physik und Astronomie, Technische Universität Berlin, 10623 Berlin, Germany.; ^3^ Laboratoire d’Acoustique de l’Université du Mans (LAUM), UMR CNRS 6613, Institut d’Acoustique-Graduate School (IA-GS), Le Mans Université, Le Mans, France.; ^4^ Experimental Physics 6 and Würzburg-Dresden Cluster of Excellence ct.qmat, Julius-Maximilians-Universität Würzburg, 97070 Würzburg, Germany.; ^5^ Research Center Future Energy Materials and Systems, Technische Universität Dortmund, 44227 Dortmund, Germany.

## Abstract

Optical generation of coherent transverse phonons by femtosecond light pulses is appealing for sub-terahertz high-speed active control of material properties. Lead-free double-perovskite semiconductors, such as Cs_2_AgBiBr_6_, attract particular interest in this respect due to their structural phase transition and strong carrier-lattice coupling. Here, we reveal that the giant anisotropic photostriction in halide perovskites with tetragonal crystal structure provides an efficient nonthermal tool for generating coherent transverse phonons. Using time-domain Brillouin spectroscopy, we observe transverse and longitudinal acoustic phonons with comparable amplitudes in the tetragonal phase of Cs_2_AgBiBr_6_ below the temperature of 122 kelvins, while, in the cubic phase, only longitudinal phonons are generated. The polarization of the transverse phonons is dictated by the projection of the crystal *c* axis on the surface plane, which leads to a prominent anisotropic polarization response in the detection. The generated strain pulses correspond to soft transverse acoustic eigenmodes with a strong temperature dependence of dispersion, providing an additional degree of freedom for hypersonic manipulation.

## Teaser

Femtosecond optical pulses launch coherent shear phonons in tetragonal halide perovskite via giant anisotropic photostriction.

## INTRODUCTION

Terahertz (THz) and sub-THz coherent acoustic phonons show great potential for manipulating and tailoring material properties enabling functionalities that involve photons (*1*–*6*), electrical currents (*7*, *8*) and fields (*9*), magnons (*10*, *11*), as well as plasmons (*12*). Experiments, e.g., have demonstrated that high-frequency coherent phonons can be used for generating THz electromagnetic waves (*13*, *14*). Due to their nanometer wavelength, sub-THz phonons can be used for nanoimaging (*15*). Ultrafast spectroscopy of coherent acoustic phonons uses excitation and detection by short femtosecond laser pulses in pump-probe techniques, representing a powerful method to investigate the lattice dynamics in a wide material spectrum (*16*, *17*). Typically, the generation of coherent phonons exploits excitation of ultrafast stress associated with the thermoelastic coupling in metals (*16*–*19*) or with the electronic deformation potential in semiconductors (*16*, *20*, *21*). In most settings, the phonons are generated with polarization vector U parallel to the propagation direction and, correspondingly, to the phonon wave vector Q , i.e., these are longitudinal acoustic (LA) phonons. However, experiments with coherent phonons, whose polarization is perpendicular to Q , i.e., transverse acoustic (TA; shear) phonons, are also in high demand for manipulation. TA phonons have a smaller sound velocity compared to LA phonons and, correspondingly, a smaller wavelength for the same frequency, which is of great advantage in imaging. Moreover, TA phonons have two independent polarization components and could be used to rotate the spins of charge carriers by chiral acoustic waves (*22*).

Generation of TA phonons requires a shear perturbation of the crystalline lattice. The most common method to induce shear strain in a material is to excite a crystalline surface of low symmetry by an ultrafast optical pulse (*23*–*25*). In this case, even isotropic compressive stress results in both compressive and shear dynamical strain and leads to the generation of quasi-LA and quasi-TA phonons with wave vectors Q perpendicular to the optically excited surface. The piezoelectric mechanism of phonon generation also may gain substantial importance (*26*, *27*). Usually, the amplitude of the generated TA phonons is much smaller than that of the LA phonons. Only recently, a few studies have reported TA phonon amplitudes comparable with those of the LA phonons (*28*–*30*). In ferroelectric BiFeO_3_, the giant anisotropic expansion was argued to explain the large amplitudes of the coherent TA phonons due to the inverse piezoelectric effect (*28*). This explanation fails obviously in centrosymmetric halide perovskites, despite recent reports of strong coherent TA phonon signals in MAPbI_3_ (*29*, *30*). Thus, the mechanisms of efficient generation of shear acoustic waves are still under debate. Material systems, such as the perovskites exhibiting a variety of structural phase transitions, provide a rich testbed for exploring previously unidentified mechanisms of TA phonon generation. Furthermore, semiconductor perovskites are highly interesting for photoinduced mechanisms as the interaction of optically excited carriers with the crystal lattice shows considerable strength compared to III-V semiconductors, for example.

In recent years, the interest in perovskite semiconductors has grown rapidly due to their success in photovoltaic applications (*31*). Inorganic lead-free double perovskites are particularly relevant here as nontoxic and stable material platform, but they, so far, suffer from inefficient charge generation and transport, for which the responsible mechanisms are yet not well understood (*32*, *33*). For the observed complex phenomena, structural phase transitions (cubic to tetragonal) (*34*) and strong electron-phonon interactions (polaron effects) (*32*, *35*), both of which can significantly influence the mobility of charge carriers (*36*), are of high relevance.

For ultrafast acoustics, double-perovskite semiconductors are particularly exciting. First, the elastic constants are about three times smaller as compared to materials such as GaAs (*37*). A soft crystal lattice allows one to achieve a significantly larger deformation for the same magnitude of stress. Second, despite the absence of the piezoelectric contribution leading to a strong renormalization of the photoinduced stress in ferroelectric perovskites (*38*), in lead halide perovskites, an analogous renormalization due to a structural phase transition is present (*39*). Calculations based on density functional theory (DFT) in (*38*) demonstrate that the photostriction in ferroelectrics has two contributions to the total energy. The first originates from the deformation potential, while the second arises from the piezoelectric effect. For this analysis, the standard Landau model was extended to account for the effects of a carrier concentration (*39*). Nonferroelectric inorganic halide perovskites exhibit two competing phase transitions (*40*): an antiferrodistortive transition (from cubic to tetragonal) and a transition to an antiferroelectric orthorhombic phase. The Landau model describes these transitions well for the exemplary material SrTiO_3_ (*41*, *42*) and gives good agreement with recent calculations based on DFT and molecular dynamics (*43*–*45*). However, the influence of excess carriers on phase stability and photostriction, as demonstrated in (*39*), has remained poorly understood. The microscopic nature of the cubic-to-tetragonal phase transition in double perovskites is analogous to that of the antiferrodistortive transition in lead halide perovskites (*44*), so that a large anisotropy of the photostrictive response is expected. Thinking about applications, the ease of synthesis and the rapid development of growth technology of perovskite semiconductors underline the potential for deposition of thin films on top of other materials, which could subsequently lead to the realization of optically driven phononic transducers (*46*).

Here, we reveal and elaborate previously unidentified mechanism for efficient optical generation of shear strain in perovskite semiconductors via the giant anisotropic photostriction of the tetragonal crystal lattice. Coherent TA phonons with amplitudes comparable to the LA modes are observed in the tetragonal phase of a Cs_2_AgBiBr_6_ crystal using time-domain Brillouin spectroscopy after resonant exciton injection by femtosecond laser pulses. We demonstrate that only one of the two TA modes is excited via the anisotropic photostriction whose polarization direction is given by the projection of the *c* axis on the sample surface. This mode corresponds to a soft TA phonon with a pronounced temperature dependence of the sound velocity in the vicinity of the structural phase transition occurring at a temperature of about 122 K, as confirmed by both time-resolved and continuous-wave (cw) Brillouin light scattering (BLS). Our results show that perovskite semiconductors and, in particular, lead-free double perovskites represent an attractive system for implementing active hypersonic devices, in which frequency and polarization of the acoustic phonons can be tuned across a wide range.

## RESULTS

### Ultrafast optical response in Cs_2_AgBiBr_6_

**Fig. 1.**
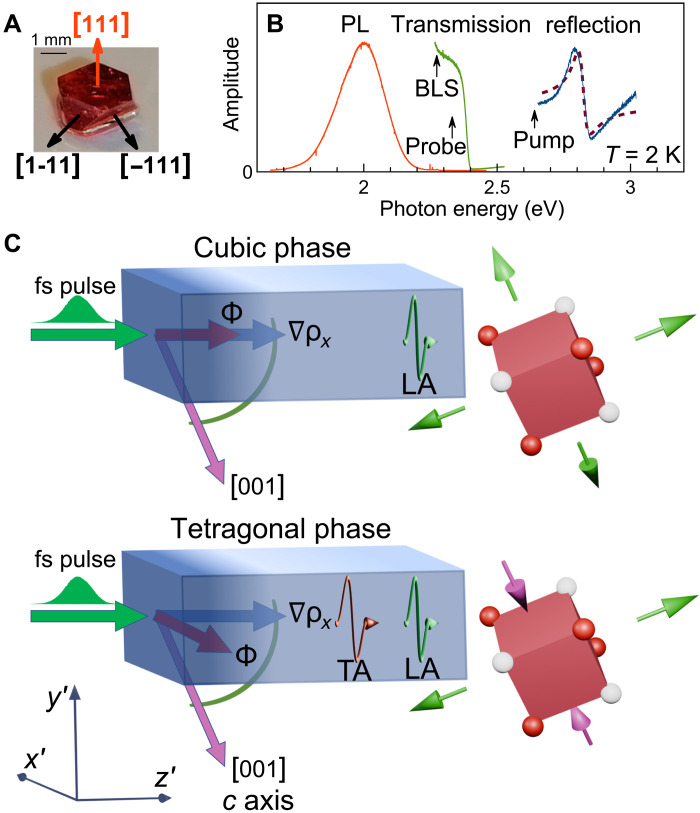
Ultrafast generation of strain pulses. (**A**) The single Cs_2_AgBiBr_6_ crystal under study. Arrows point along the main crystallographic directions of the facets. (**B**) Reflectivity (blue), transmission (green), and photoluminescence (PL; red) measured at *T* = 2 K. The reflectivity spectrum shows the exciton resonance at 2.8 eV. The dashed line is a fit to the reflectivity spectrum as described in the text. The transmission spectrum is measured in the vicinity of the absorption edge. Vertical arrows indicate the photon energies in the time-resolved pump-probe and cw BLS experiments. (**C**) Schematic presentation of the excitation of a strain pulse by a femtosecond (fs) optical pump pulse along the $z^{\prime }$ direction. The color gradient indicates the surface layer containing photoexcited carriers. In the cubic phase, the photogenerated stress driving force $\Phi \;∥\;z^{\prime }$ results in compressive strain only, so that a LA phonon pulse is launched in this case, as shown by the green wiggly arrow. In the tetragonal phase, the expansion coefficient along the [001] direction (*c* axis) is different from that along the [100] and [010] directions. Then, the driving force Φ acquires an in-plane component (along $y^{\prime }$ ) and leads to shear strain. If the expansion coefficients have different signs (see green and magenta arrows), then the shear strain amplitude is large, giving rise to TA and LA pulses of comparable amplitudes (red and green wiggly arrows).

Single crystals of Cs_2_AgBiBr_6_ were grown from a mixture of CsBr, AgBr, and BiBr in a 2:1:1 molar ratio in 48 wt % HBr (*47*, *48*). The reaction flask was heated to 110°C for complete dissolution of the metal salts and cooled down at a rate of 1°C/hour to room temperature (see Materials and Methods). The simple cubic crystal structure in ambient conditions was proven by powder x-ray diffraction (XRD; see section S1). The crystal facets are associated with the (111) or equivalent crystallographic planes as shown in Fig. 1A. Note that, in the tetragonal phase, these planes have the Miller indices (011) and equivalent.

The optical properties of the investigated sample are summarized in Fig. 1B. The low-temperature reflectivity spectrum (blue line) shows a strong feature centered at about 2.8 eV, which is associated with the exciton resonance corresponding to the direct interband optical transition at the Γ-point (*35*). Fit of the reflectivity spectrum with the dielectric function $\epsilon \left(h\nu \right)=\epsilon_{0}\left[1+\frac{\Delta_{\text{LT}}}{h\left(\nu -\nu_{0}\right)+i\Gamma_{X}\;/\;2}\right]$ gives the oscillator strength with the longitudinal transverse splitting $\Delta_{\text{LT}}=24$ meV, full width $\Gamma_{x}=43$ meV, and the exciton resonance energy $h\nu_{0}=2.8$ eV, where hν is the photon energy and $\epsilon_{0}=4$ is the background dielectric constant taken from (*49*). The corresponding absorption length is estimated to be in the order of 100 nm. The green and red lines in Fig. 1B show the transmission and photoluminescence (PL) spectra with a sharp absorption edge at about 2.4 eV and a broad PL peak at about 2.0 eV, in agreement with previous studies (*35*, *36*). Note that these values cannot be directly attributed to the indirect bandgap due to the strong electron-phonon coupling with a Huang-Rhys factor in the order of 10 (*35*).

For investigation of the optically generated strain pulses, we use the established method of time-domain Brillouin spectroscopy (*27*). As shown in Fig. 1C, the pump pulse with a photon energy above the perovskite absorption edge generates photoexcited carriers in the thin surface area of the crystal, which leads to the formation of a stress driving force Φ and to subsequent propagation of a strain pulse away from the surface along the $z^{\prime }$ direction, i.e., along the gradient of the photoexcited carrier density $\nabla \rho_{X}$ . In isotropic or cubic crystals with high symmetry crystallographic directions, $\Phi \;∥\;z^{\prime }$ so that only longitudinal strain pulses are generated. However, as will be shown below, anisotropic expansion and, in particular, simultaneous expansion and compression along different directions can also induce a driving force with a direction different from $z^{\prime }$ , which subsequently leads to the generation of shear strain pulses (TA pulse in Fig. 1C).

Time-resolved pump-probe measurements were performed using two different laser repetition frequencies of 80 MHz and 30 kHz, which are below referred to as high (HF) and low (LF) frequency, respectively. In the HF case, the energy fluence of the pump laser $\Psi_{\text{pump}}$ is weak amounting to about 30 μJ/cm^2^, while, for the LF measurements, $\Psi_{\text{pump}}$ ~ 700 μJ/cm^2^ is significantly stronger. The duration of the laser pulses is about 150 fs (see Materials and Methods).

**Fig. 2.**
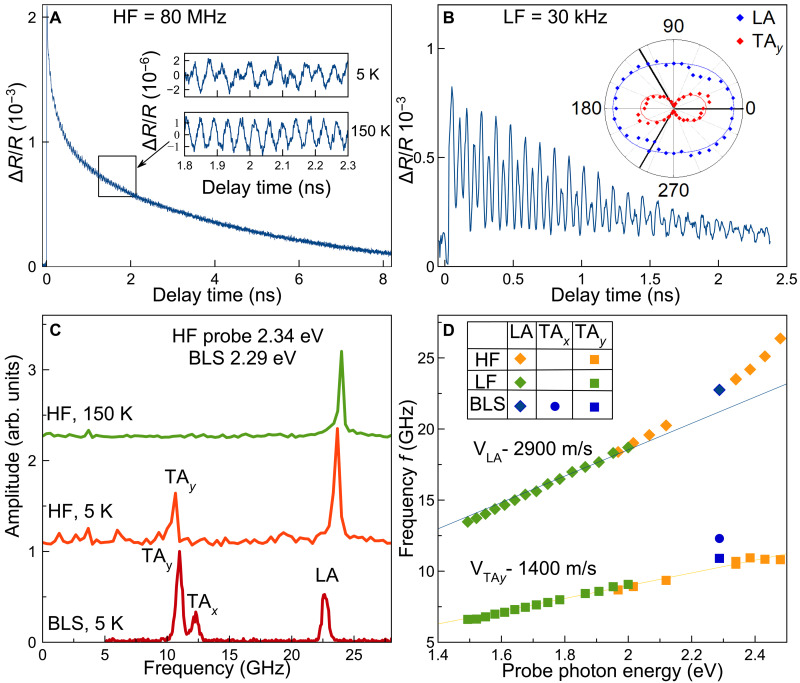
Dynamics of pump-induced reflectivity modulation. (**A** and **B**) Examples of pump-probe transients of differential reflectivity ΔR/R from the Cs_2_AgBiBr_6_ crystal at *T* = 5 K, i.e., in the tetragonal phase, measured along the crystal direction [011] ([111] in the cubic phase) in HF and LF experiments with repetition frequencies of 80 MHz and 30 kHz, respectively. In (A), the photon energies of the pump $h\nu_{\text{pump}}$ and probe $h\nu_{\text{probe}}$ pulses are set to 2.638 and 2.339 eV, respectively. The insets show fast oscillatory signals below and above the structural phase transitions, taken at *T* = 5 and 150 K. In (B), $h\nu_{\text{pump}}=2.818$ eV and $h\nu_{\text{probe}}=2.0$ eV. The inset shows a polar plot of the amplitudes of the fast oscillatory components with frequencies $f_{\text{LA}}=18.6$ GHz and $f_{{\text{TA}}_{y}}=9$ GHz (blue and red diamonds, respectively) as function of the direction θ of the probe pulse polarization relative to the $y^{\prime }$ axis. The solid black lines show possible projections of the directions of the *c* axis on the crystal surface. The solid red and blue lines are fits using $A-B{\text{sin}}^{2}\left(\theta \right)$ . (**C**) The top and middle curve correspond to FFT spectra of the pump-probe signals in (A), measured at 5 K (red) showing two peaks in the tetragonal phase and measured at 150 K (green) with only one peak in the cubic phase. The lower spectrum corresponds to cw BLS measured at the photon energy $h\nu_{\text{BLS}}=2.287$ eV for *T* = 5 K. (**D**) Frequencies of the FFT peaks of the coherent longitudinal LA (diamonds), transverse acoustic TA*_x_* (circles), and TA*_y_* (squares) phonons as function of the probe photon energy. The cw BLS data are shown by the blue symbols, the LF data by the green symbols, and the HF data by the yellow symbols. Linear fits through zero frequency are presented by lines.

Exemplary pump-probe results are summarized in Fig. 2, namely, examples of differential reflectivity ΔR/R transients at *T* = 5 K temperature using HF and LF excitation in Fig. 2 (A and B), respectively. In all cases, the signal can be described by fast oscillations superimposed on a slowly varying decaying transient. The latter originates mainly from carrier relaxation after pulsed photoexcitation, which depends on the energy of the laser pulse, i.e., varies for HF and LF excitation. For HF excitation, we observe a biexponential decay with characteristic times of 0.3 and 4 ns, while, for the LF excitation, a single exponential decay with about 2-ns decay time is found. We note that the relative magnitude of the oscillatory signal is much more pronounced for LF excitation, which is due to the larger energy in an excitation pulse. In what follows, we focus on the fast oscillatory signal to be analyzed after subtraction of the slowly varying exponential signal, as shown in the inset of Fig. 2A for 5- and 150-K temperature.

Unexpectedly, at the low temperature of *T* = 5 K, when the crystal is in the tetragonal phase, the signal contains two frequencies $f_{i}$ of about 10 and 23 GHz, see Fig. 2C, giving normalized fast Fourier transform (FFT) spectra of the oscillatory signals shown in Fig. 2A for HF excitation. At temperatures above $T_{c}\approx 122$ K, the crystal structure transforms into the cubic phase (*34*). Here, only the component with the higher frequency persists, see the 150-K temperature case Fig. 2C. The two frequencies are independent of the photon energy of the pump pulses but increase linearly with increasing probe photon energy $h\nu_{\text{probe}}$ , see Fig. 2D.

This behavior is typically observed in time-domain Brillouin scattering on acoustic phonons, i.e., when the probe pulse is scattered from the pump-induced strain pulse propagating away from the surface with the speed of sound $V_{i}$ (*27*). In this case, the oscillation frequency is determined by $f_{i}=2n_{r}\nu_{\text{probe}}V_{i}\;/\;c$ , where *c* is the speed of light and $n_{r}$ is the refractive index in Cs_2_AgBiBr_6_. Taking the refractive index *n*_r_ = 2 at room temperature from (*49*), we can fit the data in Fig. 2D with a linear function and estimate the speed of sound for the LA and one of the TA modes as $V_{\text{LA}}=2900$ m/s and $V_{{\text{TA}}_{y}}=1400$ m/s at *T* = 5 K. These values are close to the data evaluated by cw BLS (*50*).

We also performed cw BLS measurements on the same sample in reflection geometry along the direction normal to the surface, using a cw single-frequency laser emitting at photon energy $h\nu_{\text{BLS}}=2.287$ eV with a spectral width of about 40 neV. The spectrum taken at *T* = 5 K in the tetragonal phase is shown in Fig. 2C. It comprises three lines with frequencies of $f_{\text{LA}}=22.8$ GHz, $f_{{\text{TA}}_{x}}=12.3$ GHz, and $f_{{\text{TA}}_{y}}=10.9$ GHz, corresponding to the longitudinal and the two transverse acoustical phonons. The presence of three distinct peaks in the tetragonal phase confirms the high-quality and single-crystalline nature of the samples. For more details about the cw BLS data, see sections S2 and S3. The frequencies of the peaks are also in agreement with the photon energy dependence of the pump-probe data in Fig. 2D (see blue symbols). Thus, we attribute the higher and lower frequencies in the pump-probe transients to the LA and TA*_y_* phonons, respectively. The slight deviation from the linear dependence at photon energies close to the bandgap ( $h\nu_{\text{probe}}∼2.0-2.4$ eV) is probably related to changes in the refractive index there (see Fig. 2D).

In a nutshell, we highlight that high-frequency coherent TA phonons are observed in the tetragonal phase, along with LA phonons, in time-domain Brillouin spectroscopy. Only one of the two TA phonon modes is detected, with an amplitude comparable to that of the LA signal. In contrast, in the cubic phase, only LA phonons are generated by the pump. The coherent phonon amplitudes are independent of the polarization of the pump beam (see section S9). In contrast, the polarization dependence of the TA signal is strongly anisotropic for the probe detection, with the peak maximum obtained for the probe polarization with maximum projection on the *c* axis, see the inset in Fig. 2B. The measured angular polarization dependence follows the form $1-0.29\;{\text{sin}}^{2}\left(\theta \right)$ for the LA and $0.55-0.5\;{\text{sin}}^{2}\left(\theta \right)$ for the TA acoustic phonons. Note that, in our experiments, the *c* axis projection on the sample surface was randomly directed along one of the three directions of the facet edges after every cooling cycle from the cubic to the tetragonal crystallographic phase. That is, in the sample coordinates, the angle θ is counted from one of these directions, which are equivalent in the cubic phase. In what follows, we identify the origin of the strong TA signal in the pump-probe transients.

### Optical generation of shear strain in tetragonal phase

For analysis of the optical generation of strain pulses, one needs to consider first the eigenmodes of the acoustic waves and their polarizations. Further, we need to consider the mechanisms of conversion of the heat and the carriers generated by the incident optical pump pulse into the deformation pulse propagating through the crystal. The eigenmodes of the acoustic waves can be expressed as plane waves with polarization vectors $U^{s}$ given by

$$\left(\rho V_{s}^{2}\delta_{\mathrm{il}}-c_{\text{ijkl}}Q_{j}Q_{k}\right)U_{l}^{s}=0$$ (1)

Here, ρ is the mass density of the material, $c_{\text{ijkl}}$ are the elastic constants of the material given in section S4, Q is the direction of the phonon wave vector, $V_{s}$ is the speed of sound for the *s*th phonon mode, and $\delta_{\mathrm{il}}$ is the Kronecker delta. The solution of Eq. 1 is discussed in more detail in section S5. For a cubic system, from Eq. 1, it follows that the LA phonons are polarized along $z^{\prime }$ , while the two TA phonon modes are degenerate and their polarization vector $U^{s}$ may be chosen to point along $x^{\prime }$ and $y^{\prime }$

$$x^{\prime }\|\left[1\bar{1}0\right]\;,\;y^{\prime }\|\left[11\bar{2}\right]\;,\;z^{\prime }\|\left[111\right]$$ (2)

To understand the origin of the photoinduced shear strain in the tetragonal phase (note that, in the tetragonal phase, the Miller indices of these directions (Eq. 2) are [100] , $\left[01\bar{1}\right]$ , and [011] ), one has to distinguish two effects: (i) the change of sound velocities, which does not change the phonon polarization, and (ii) the change of phonon polarization vectors with respect to the axis frame. Calculation of the elastic constants using a DFT approach (see section S4 for details) renders the following information about polarization mode mixing: (i) for the LA mode, a ∼0.8% admixture of the TA_1_ mode and a ∼6.5% admixture of the TA_2_ mode; (ii) for the TA_1_ mode, admixtures of ∼5% of the TA_2_ mode and ∼0.4% of the LA mode; and (iii) for the TA_2_ mode, admixtures of ∼5% of the TA_1_ mode and ∼6.5% of the LA mode. This result assumes that, while the change of sound velocities (which, in particular, splits the TA mode frequencies) is large, the crystal structure effect on the polarizations may be calculated perturbatively (see section S5 for the eigenmodes in the tetragonal phase). As a result, in the tetragonal phase, the TA_1_, TA_2_, and LA phonons are predominantly polarized along the axes $x^{\prime }$ , $y^{\prime }$ , and $z^{\prime }$ , respectively. This means that the large amplitude of the shear strain observed in the experiment cannot be explained by mixing of the TA and LA modes but requires a different mechanism.

The amplitude $A_{s}$ of the generated strain pulse after laser excitation is proportional to the product of the polarization of the *s*th phonon mode $U^{s}$ and the “driving force” Φ whose components are given by (see details in section S6)

$$\Phi_{i}=\frac{\partial \Sigma_{\mathrm{ij}}}{\partial x_{j}}$$ (3)

where Σ is the tensor of stress induced by the pump laser pulse. As explained above, the direction of $U^{s}$ only slightly deviates from $x^{\prime }$ (TA_1_), $y^{\prime }$ (TA_2_), and $z^{\prime }$ (LA). This means that the amplitudes of the generated phonons are proportional to the components of the driving force Φ.

The stress tensor contains two main contributions (*51*). The first one is given by the linear expansion of the lattice induced by heat (*16*), which is proportional to the increase of the lattice temperature δT

$$\Sigma_{\mathrm{ij}}^{T}=-3\delta_{\mathrm{ij}}B\beta_{i}\delta T\left(z^{\prime }\right)\;$$ (4)

where $\beta_{i}$ is the linear expansion temperature coefficient and *B* is the bulk modulus. The other one is proportional to the density of the photoexcited carriers $\rho_{X}$

$$\Sigma_{\mathrm{ij}}^{n}=\delta_{\mathrm{ij}}B\alpha_{i}\rho_{X}\left(z^{\prime }\right)\;$$ (5)

Here, the $\alpha_{i}$ are the photostriction coefficients, i.e., the changes of lattice constant by the photogenerated electron-hole pairs. In literature, the stress due to the presence of carriers is called either “deformation potential” or “photostriction.” We use the second term to highlight that the deformation potential is not the only mechanism contributing to the phenomenon described by Eq. 5. We exclude impulsive stimulated Brillouin scattering as origin of coherent phonon generation because this mechanism leads to a strong dependence of the photoinduced strain on the polarization of the exciting light (*52*, *53*). This is in contrast to our experimental results, where the oscillatory signals are independent of the polarization state of the pump pulse (see also section S9 for details). We also do not have to consider the inverse piezoelectric effect because halide perovskites have a center of inversion.

In GaAs, the strain caused by lattice heating as described by Eq. 4 is estimated to give ∼3% of the contribution given by Eq. 5 (*51*, *54*). In MAPbI_3_, the photostriction coefficient is estimated (*55*) to be at least two orders of magnitude larger than in GaAs. Therefore, it is only consequent to expect that this contribution is the dominating one in (double) perovskites.

Referencing to the surface that is the plane with normal −n , both the temperature increase and the density of the photogenerated carriers are given by exponential distribution functions

$$\delta T\left(r\right)\;,\;\rho_{X}\left(r\right)\propto \text{exp}\left(-n\cdot r\;/\;\zeta \right)$$ (6)

where ζ is the absorption length along the coordinate r . In case of isotropic thermal expansion and photostriction, the driving force is directed along the surface normal Φ∥n so that only LA phonons can be generated.

However, for materials of lower symmetry, the linear expansion coefficient may be different for different crystallographic directions (*56*). The thermal coefficients for the linear expansion in Cs_2_AgBiBr_6_ are almost equal in magnitude but have different signs for the directions perpendicular to and along the [001] crystal axis (*34*). This behavior is also typical for the tetragonal phase perovskites: in β-MAPbI_3_ (space group #140, I4/mcm ), $\beta_{⊥c}\approx -\beta_{\|c}$ (*57*).

To our knowledge, neither measurements nor calculations of the photostriction coefficients of Cs_2_AgBiBr_6_ are available. However, calculations for β-CsPbI_3_ show that the $\alpha_{i}$ have different signs perpendicular to and along the *c* axis ( $\alpha_{⊥c}\;/\;\alpha_{\|c}\approx -0.6$ ) (*39*), similar to the temperature expansion coefficient. In CsPbI_3_, this giant anisotropy has been qualitatively explained by accounting for the cubic-to-tetragonal phase transition (*38*, *39*). Recently, it also has been demonstrated that one of the main contributions to the exciton-phonon interaction in CsPbI_3_ is associated with the phonon mode that is responsible for the cubic-to-tetrahedral phase transition: rotation of octahedra formed by halide atoms in (001) plane (*58*). Therefore, we expect that a similarly giant anisotropic photostriction is present in double perovskites showing a cubic to tetragonal phase transition and strong electron-phonon interaction.

**Fig. 3.**
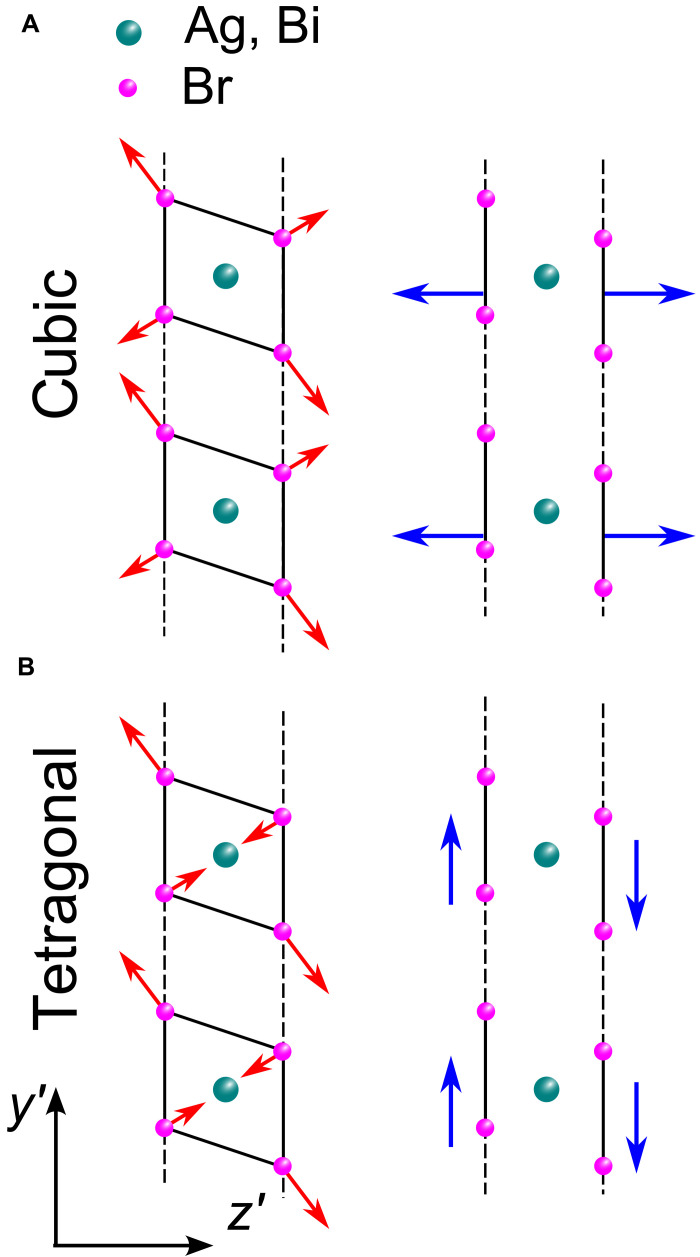
Shear strain generation. Sketch illustrating the occurrence of a transverse component of the driving force in the tetragonal phase (**B**) in comparison to the cubic phase (**A**). The colored spheres show the positions of the atoms, the red arrows indicate the direction of the force acting on the individual atoms, and the blue arrows show the average stress force acting in the surface plane. The red arrows with shorter lengths result from the projection of the stress vector into the $y^{\prime }z^{\prime }$ plane. They have to be taken into account twice due to the two atoms in the cell along the $x^{\prime }$ axis.

As a result of the anisotropy, the driving force acquires an in-plane component if the crystal *c* axis is neither parallel to the sample surface nor normal to it. Note that this does not require excitation of a surface of low symmetry. For example, in our case, this “high symmetry” condition is fulfilled for optical excitation of the $x^{\prime }y^{\prime }$ facet. The deviation of $\alpha_{⊥c}$ from $\alpha_{\|c}$ results in rotation of the driving force Φ in the $y^{\prime }z^{\prime }$ plane, see Figs. 1C and 3. The $y^{\prime }$ component of Φ generates TA_2_ phonons. For $\alpha_{⊥c}≃-\alpha_{\|c}$ , the amplitude of the generated TA_2_ phonons is $2\sqrt{2}$ times larger than the amplitude of the generated LA phonons. Note that TA_1_ phonons generally could also be generated because the elastic constant $c_{16}$ is nonzero in double perovskites. This is in contrast to the tetragonal phase in lead halide perovskites, where this process is forbidden by symmetry ( $c_{16}=0$ ). Nevertheless, according to our experiments on Cs_2_AgBiBr_6_, only one of the TA phonon modes is excited, which can be explained by the small magnitude of $c_{16}$ . Hence, the transverse modes labeled TA*_x_* and TA*_y_* in the experimental data of Fig. 2 correspond to transverse phonons polarized along the $x^{\prime }$ (TA_1_ ≡ TA*_x_*) and $y^{\prime }$ (TA_2_ ≡ TA*_y_*) axes, respectively. This is supported by the correspondence of the polarization dependences of the cw and time-resolved BLS signals for the TA*_y_* peaks (see fig. S4). Based on this analysis, we can conclude that the TA*_y_* mode has a lower frequency than the TA*_x_* mode. As follows from Fig. 2D, the frequency of the low-energy TA phonon in the cw BLS spectra (blue square) coincides with the only mode (TA*_y_*) excited in the pump-probe experiment (yellow squares).

The detected signal arises from reflection of the probe pulse at the strain wave. Its strength is given by the strain-induced variation of the dielectric constant $\Delta \hat{\varepsilon }$ (*24*, *59*), which, in turn, is proportional to the strain tensor $\hat{\epsilon }$ (*60*–*62*). As shown in section S5, the strain tensor for different modes is proportional not only to the amplitude $∼\Phi \cdot U^{s}$ but also to $\epsilon_{\mathrm{ij}}^{s}=\frac{1}{2}\left(U_{i}^{s}Q_{j}+U_{i}^{s}Q_{i}\right)$

For the general photoelastic tensor with tetragonal symmetry, all relevant components of the dielectric constants may be nonzero, leading to light scattering on all possible phonons resulting in a complex polarization dependence, see section S7 for details. In the opposite limiting case of a fully isotropic material, the probe signal would be reflected only from the LA strain pulse, independent of its polarization, and proportional to $p_{1122}$ . None of these scenarios is found in our experiment. We remind that it is necessary to account for both the LA and TA*_y_* strain pulses as the ones predominantly excited by the pump laser pulses. As follows from eq. S29, the polarization dependence of the scattered optical field contains both zeroth- and second-order harmonic contributions. From fitting the data in the inset of Fig. 2B, we find that the scattering of the probe from LA phonons is mainly governed by the zeroth-order harmonic (blue ellipsoid), whereas, for the TA*_y_* phonons, it is dominated by the second-order harmonic (two-lobed red rosette). According to the theoretical analysis in section S7, this means that the opto-elastic tensor is almost isotropic in the plane normal to the *c* axis, and $p_{1122}\approx p_{1133}$ . We emphasize that the strong polarization dependence of the scattered probe beam provides a simple optical tool for determining the direction of the *c* axis in the tetragonal crystal phase.

### Temperature dependence: Tetragonal vs. cubic phase

**Fig. 4.**
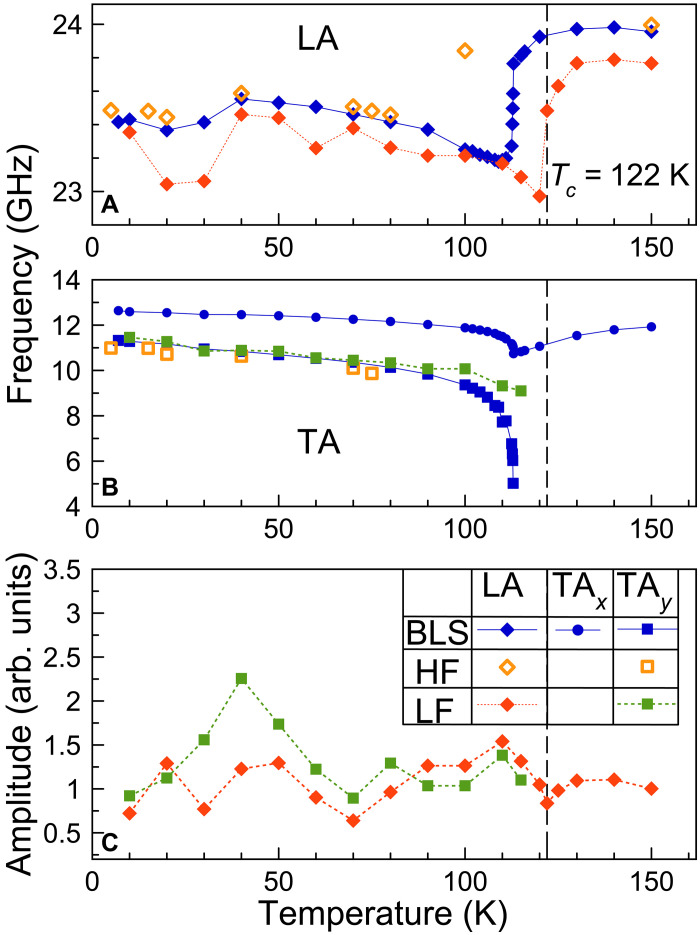
Temperature dependence of the phonon frequencies. The data are shown by orange symbols for the HF pump-probe, by green and red symbols for the LF pump-probe, and by blue symbols for the cw BLS spectroscopy (recalculated for the photon energy of hν=2.34 eV). (**A**) Diamond symbols correspond to the LA phonons, (**B**) squares to the TA*_y_* phonons, and circles to the TA*_x_* phonons. The vertical dashed line corresponds to the phase transition temperature *T_c_* = 122 K. (**C**) Temperature dependence of the signal amplitude for the LA and TA*_y_* phonons measured in the LF pump-probe experiment.

The temperature dependence of the phonon frequencies and their amplitudes are particularly interesting because of the crystal phase transition from tetragonal to cubic phase. The corresponding data are summarized in Fig. 4. The phonon frequencies evaluated from cw BLS and LF pump-probe data are recalculated for the same probe photon energy of hν=2.34 eV, used in the HF pump-probe measurements. Here, we assume a linear dependence $f_{i}\left(h\nu \right)$ on probe photon energy, as follows from Fig. 2D. This photon energy corresponds to maximum oscillatory signal strength close to the absorption edge, see Fig. 1B.

The cw BLS data for the three acoustic phonon frequencies demonstrate sharp changes for temperatures around the structural phase transition at $T_{c}∼122$ K. For the longitudinal mode we observe a frequency increase from 23.4 GHz by about 0.5 GHz to almost 24 GHz with increasing temperature across the tetragonal to cubic phase transition. The frequency of the TA*_x_* phonon undergoes a small decrease right below the phase transition, while, above $T_{c}$ , it recovers to a similar value as in the tetragonal phase. We observe a strong softening of the TA*_y_* mode in the tetragonal phase when approaching the phase transition. The TA*_y_* peak decreases from 11 to 5 GHz accompanied by the formation of a broad band with almost zero offset (see section S2 for cw BLS spectra around the phase transition). In the cubic phase, the TA modes are degenerate so that only one peak at ∼12 GHz for 150 K is observed in the BLS spectrum.

The difference in the temperature, at which the phase transition occurs in the cw and time-domain BLS, is attributed to local heating of the sample by the laser, which is most pronounced for HF excitation with high integrated power (2 kW/cm^2^) so that the actual crystal temperature within the excitation spot is about 40 K higher than the bath temperature. For cw BLS, the excitation power is even higher (100 kW/cm^2^), but the photon energy of 2.287 eV is below the direct bandgap and the corresponding increase of temperature is only about 10 K. This is in contrast to the negligible heating observed in the LF pump-probe data with a time-integrated power density of about 20 W/cm^2^, for which the phase transition temperature corresponds to the value of 122 K reported in literature (*34*).

As discussed previously, only the LA and TA*_y_* phonons are detected in the tetragonal phase by time-domain BLS, while, for $T>T_{c}$ in the cubic phase, only the LA coherent phonons are present in the spectrum. The temperature dependence of the phonon frequencies is in correspondence with that obtained in cw BLS spectroscopy, i.e., we observe hardening of the LA mode by 0.5 GHz and TA*_y_* mode softening from 11 to 9 GHz in the vicinity of the phase transition (see Fig. 4). Accordingly, the TA*_y_* mode softening allows one to tune the speed of sound of the photoinduced shear strain pulse by temperature.

The temperature dependence of the LA and TA*_y_* peak amplitudes in the FFT spectra is shown in Fig. 4C. For the LA peak, we observe small variations of the magnitude in the ±20% range of fluctuations mainly attributed to inhomogeneities of the sample. The TA phonon signal shows a nonmonotonic behavior with a maximum around 40 K, where the amplitude of the TA*_y_* peak is twice larger as compared to the LA amplitude. Apart from the feature at 40 K, the TA*_y_* peak amplitude follows closely the temperature dependence associated with the LA phonons and vanishes after transition to the cubic phase. The temperature dependence of the photoelastic constants in the transparency range ( hν=2 eV) is expected to be weak, and, therefore, we assume that the amplitudes of the FFT peaks follow the amplitudes of the corresponding photogenerated strain pulses. This implies that the photoinduced strain contains a substantial nonthermal contribution as discussed in the following section.

## DISCUSSION

To summarize, we have revealed and elaborated the mechanism for efficient generation of shear strain in the tetragonal phase of lead-free Cs_2_AgBiBr_6_ double-perovskite single crystals. The mechanism manifests in (i) generation of coherent TA phonons with polarization dictated by the orientation of the *c* axis, (ii) strong polarization dependence in the optical detection by the probe, and (iii) observation of soft TA phonons with a strong frequency dependence at temperatures around the structural phase transition. The latter can be useful for tuning the efficiency of stimulated Brillouin scattering on phonons with particular frequencies, if they are generated by lasers with high repetition frequency rates, e.g., 1 and 10 GHz as demonstrated for resonant excitation of magnons (*63*, *64*). The mechanism of shear strain generation is based on the giant anisotropic photostriction of the crystal lattice so that it should be present in a large variety of perovskite materials with tetragonal crystal symmetry, e.g., in MAPbI_3_ at room temperature. In this respect, halide perovskite semiconductors are highly promising materials, allowing one to choose the structural phase, the band structure (direct or indirect), and the strength of electron-phonon coupling. For future comparative investigations, Table 1 gives a list of different halide perovskite compounds exhibiting tetragonal crystal structure for which inspection of electron-phonon interaction effects is highly interesting.

**Table 1.** Parameters of different halide perovskite compounds exhibiting a tetragonal crystal structure.

Substance	Direct/indirect bandgap, eV	Temperature range with tetragonal crystal structure, K	Huang-Rhys factor
MAPbI_3_ (*55*, *70*)	Direct, ∼1.6 eV	162–327	1–1.5
MAPbBr_3_ (*70*, *71*)	Direct, 2.32 eV	145–237	0.5–1.5
CsPbBr_3_ (*70*–*72*)	Direct, 2.36 eV	361–403	12
CsPbCl_3_ (*70*, *73*, *74*)	Direct, 2.98 eV	310–320	1.34–3.38
Cs_2_AgBiBr_6_ (*34*, *35*)	Indirect, ∼2.0 eV	Below 122	11.7
Cs_2_NaBiCl_6_ (*75*–*77*)	Indirect, ∼3.4 eV	Below 90	7.1
Cs_2_KInCl_6_ (*78*, *79*)	Indirect, 2.84 eV	–	39

The weak temperature dependence of the photogenerated compressive strain, carried by coherent LA phonons, along with the nonmonotonic behavior of the shear strain amplitude associated with coherent TA phonons, provides evidence for the nonthermal nature of the effect, i.e., for the direct generation of strain via carrier-phonon interaction that is accomplished due to the anisotropic photostriction. The heat capacity of Cs_2_AgBiBr_6_ has a strong nonlinear *T*-dependence, which has a sharp increase below 70 K and a slow variation at higher temperatures, while the thermal expansion coefficient does not depend on temperature (*34*). As shown in section S8, this means that an increase of the temperature due to laser pulsed excitation should be at least twice more efficient at low temperatures than at high temperatures so that one would expect a monotonic decrease of the signal amplitude with temperature increase in case of a photoinduced thermal expansion. Thus, the dominant effect cannot be associated with the temperature-based mechanism (see Eq. 4) but occurs mainly due to the photostriction given by Eq. 5. Assuming that the photostriction mechanism is at least as strong as that induced by temperature variations and that the strain field amplitude is on the order of $10^{-3}$ for our experimental conditions, we can estimate the photostriction coefficients $\alpha_{i}$ . Using the exciton density $\rho_{X}\left(0\right)∼10^{20}$ cm^−3^ near the surface, we obtain $|\;\alpha_{i}\;|∼10^{-23}$ cm^3^.

We stress that the giant anisotropic photostriction is directly related to the strong interaction of excitons with optical phonons, which gives rise to a set of peculiarities compared to conventional semiconductors (*65*). In particular, exciton energy is related to the rotation of octahedra, formed by the halide atoms, around the *c* axis, that significantly influences the relative expansion along different axes of the unit cell. Note that such a rotational phonon mode is also associated with the crystallographic phase transition between cubic and tetragonal phases (*58*). As shown above, giant anisotropic expansion transforms in the coordinate system of the sample to the generation of strong shear strain. Therefore, the coherent excitation of soft TA phonons could be due to the interaction with the specific optical phonon eigenmodes responsible for the tetragonal to cubic phase transition. We emphasize that, at T≈40 K, the TA*_y_* signal reaches maximum with an amplitude twice larger than that of the LA phonons. Notably, at this temperature, Raman spectroscopy has revealed important modifications in the optical phonon spectrum (*66*). This observation suggests a possible correlation with the efficient excitation of coherent transverse phonons mediated by the electron-phonon interactions and calls for future investigations of the underlying excitation mechanism. Further studies of the microscopic origin of the giant anisotropic photostriction could allow one to adjust the parameters of the material for efficient photoinduced generation of shear strain and realization of perovskite-based phononic transducers, also for chiral phonons.

## MATERIALS AND METHODS

### Sample growth

Single crystals of Cs_2_AgBiBr_6_ were grown by controlled cooling following the protocol in (*47*, *48*). CsBr (1.5 mmol, 319.21 mg, 2 equiv), AgBr (0.75 mmol, 140.83 mg, 1 equiv), and BiBr_3_ (0.75 mmol, 336.52 mg, 1 equiv) were mixed in a 25-ml vial, followed by the addition of 10 ml of 48 wt % HBr. The reaction was heated in a silicon oil bath to 110°C during 20 min and kept at this temperature for 4 hours to obtain a completely dissolved precursor solution. Next, the solution was cooled down at a rate of 1°C/hour to room temperature to obtain single crystals of ~4 mm size. The crystals were then removed from the mother liquor, dried with paper tissue, and rinsed with dichloromethane. Single crystals were grinded into powder before XRD measurements.

### Sample characterization

XRD measurements were carried out using a General Electric XRD 3003 TT diffraction system using a Cu-K*_α_* radiation source with a wavelength λ of 1.5406 Å ( V=40 kV, I=40 mA), in ambient conditions. The resulting XRD pattern of Cs_2_AgBiBr_6_ matches the one simulated for a cubic lattice (see fig. S1) (*67*). Based on the shape of the single crystal, one can unambiguously conclude that the facets are formed by either {111} or {101} crystal planes (see Fig. 1A). From cw BLS measurements at room temperature, we identified that these facets correspond to the {111} orientation, as both the LA and the doubly degenerate TA peaks are present. Note that, in the case of {101} orientation, only the LA phonon is observed in accordance with the selection rules.

### PL spectroscopy

The PL spectra were measured at *T* = 2 K in a bath cryostat with the sample immersed in superfluid helium. The photon energy for excitation was 3.493 eV. A halogen lamp was used as a source of white light for reflection measurements.

### Time-domain and cw Brillouin spectroscopy

In the pump-probe and cw BLS studies, the sample was cooled down in a helium flow cryostat, allowing the use of microscope objectives with working distance of >10 mm. In all experiments, a 20× microscope objective with a numerical aperture of 0.4 was used. The beams were focused into spots of about 5 μm diameter.

cw BLS was measured using a stabilized double tandem Fabry-Pérot Brillouin spectrometer (TFP-2 from Table Stable). The distance between the mirrors is set to 3 mm, and the width of the entrance and exit slits was 300 μm. For excitation, we used a single-frequency cw laser with the photon energy of *2.*287 eV (542-nm wavelength) and a spectral width below 10 MHz. The sample was excited at the laser power of 20 mW in backscattering geometry with nominally normal incidence (unless specified otherwise). The scattered signal was detected in a copolarized scheme, corresponding to the detection of vertically polarized backscattered light.

Time-domain Brillouin spectroscopy was performed using a transient pump-probe technique at two different repetition frequencies of 80 MHz and 30 kHz. Pump-probe at 80 MHz was implemented using an asynchronous optical sampling technique with two laser sources tunable in the range of 470‐to700‐nm wavelength (Toptica FFpro). The repetition rates of the lasers were synchronized with an offset frequency equal to δf=2 kHz. The sample was excited by both lasers serving as pump and probe, respectively, using the same microscope objective in confocal reflection geometry. The reflected probe beam was detected using a fast photodiode in combination with a digitizer card, giving an overall time resolution better than 10 ps. The energy fluence of the pump and probe pulses Ψ were set to 30 and 10 μJ/cm^2^, respectively.

Time-domain Brillouin spectroscopy at 30 kHz was performed using an optical parametric amplifier system tunable in the range of 0.4‐to2‐μm (PHAROS Femtosecond Lasers). A delay line was implemented using a retroreflector mounted on a motorized linear stage. The intensity of the probe beam was modulated with a chopper at the frequency of 1 kHz, and the reflected probe beam was detected with a photodiode in conjunction with a lock-in amplifier. The energy fluences of the pump and probe pulses Ψ were set to 0.7 and 3.5 mJ/cm^2^, respectively. The fluence of the probe pulse exceeded, therefore, that of the pump pulse, but its photon energy was set to below the energy gap ( $h\nu_{\text{probe}}<2$ eV), so that the probe beam was subject to negligible absorption that could influence the density of photoexcited carriers.

The duration of the pulses in both pump-probe setups was about 150 fs. The angular dependence of polarization in Fig. 2B was taken for linearly copolarized pump and probe beams. The polarization of the beams was set using Glan polarizers. The polarization rotation was achieved using a half-wave plate in front of the microscope objective that corresponds to a rotation of the sample with respect to the laboratory frame.

The crystal spot undergoes damage under laser excitation at an energy fluence exceeding 1 mJ/cm^2^ for 30-kHz excitation and above 50 μJ/cm^2^ for 80-MHz excitation at 5 K in the tetragonal phase. The former is related to the energy per pulse, while the latter is governed by the total (average) power accumulated in the excitation spot.

### DFT calculations

The DFT calculations were performed using the WIEN2k package (*68*). For the calculation of the elastic tensor components we use the IRelast package (*69*). First, the structure is optimized using the PBEsol exchange-correlation functional. Next, for a few selected deformations, the energy as function of the deformation amplitude is calculated, and, from the fit of the calculated energy, the combinations of elastic tensor components are extracted.

## Supplementary Materials

### This PDF file includes:

Sections S1 to S9 Figs. S1 to S6 References
