# Supplementary material for: Efficient launching of shear phonons in photostrictive halide perovskites
Source: Sci Adv. 2025 Nov 5;11(45):eadw9172. doi: 10.1126/sciadv.adw9172 (PMC13141899; doi:10.1126/sciadv.adw9172)
Supplement: Supplementary file 1 — Sections S1 to S9 Figs. S1 to S6 References [file sciadv.adw9172_sm.pdf]

Science Advances
AAAS

Supplementary Materials for

Efficient launching of shear phonons in photostrictive halide perovskites

Dmytro O. Horiachyi *et al.*

Corresponding author: Dmytro O. Horiachyi, dmytro.horiachyi@tu-dortmund.de;
Mikhail O. Nestoklon, mikhail.nestoklon@tu-dortmund.de

Sci. Adv. **11**, eadw9172 (2025)
DOI: 10.1126/sciadv.adw9172

This PDF file includes:

Sections S1 to S9
Figs. S1 to S6
References

S1 X-ray diffraction

The results of X-ray diffraction measurements and corresponding simulations for grained $Cs_2AgBiBr_6$ crystals are shown in Fig. S1.

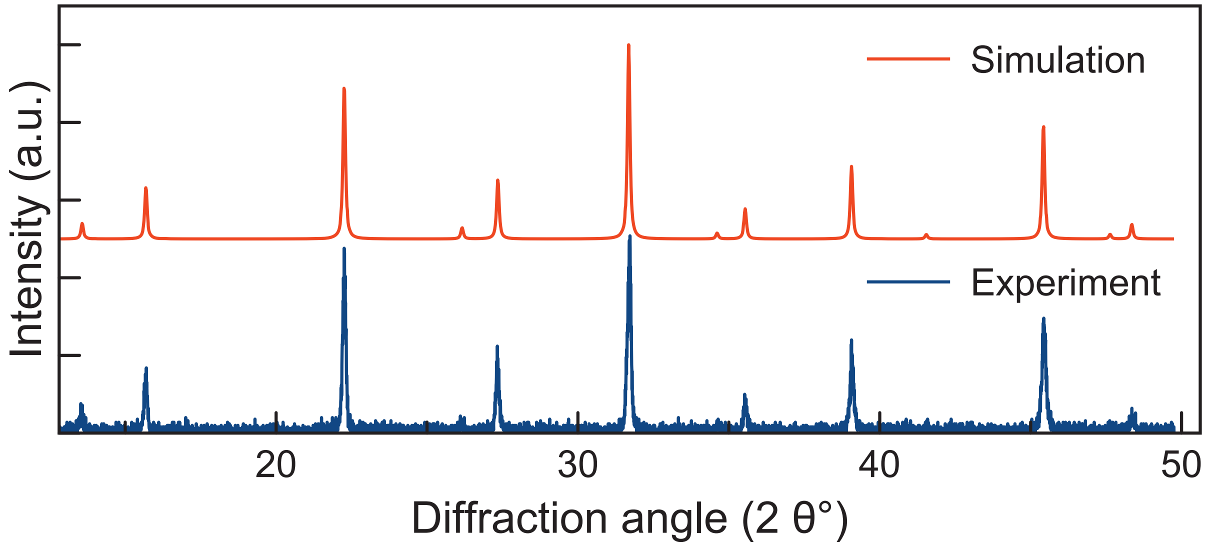

Figure S1: **XRD powder pattern of a grained $Cs_2AgBiBr_6$ crystal measured at room temperature.** The peaks can be assigned to a simple cubic crystal structure (*47*). The simulated XRD powder pattern data for the cubic lattice were calculated using the Mercury CCDC software with the crystallographic data from (*72*).

S2 Continuous wave Brillouin light scattering spectroscopy

Here, the frequencies of the BLS peaks measured at $T = 5$ K in reflection geometry for two geometries, normal light incidence and 45° light incidence with respect to the $x'y'$ sample surface, are compared. The results are presented in Fig. S2. The change of the incidence angle in reflection geometry leads to a change of the wavevector of the phonon involved in the scattering $q \approx 2k_{in} \sin(\Theta/2)$ with $k_{\mathrm{in}} \approx k_{\mathrm{out}}$, where k_{in} and k_{out} are the wavevectors of the incident and scattered light, and the angle $\Theta = \pi - \arcsin(\sin(\gamma)/n_r)$ is determined by the incidence angle γ. Note that in this case the direction of the wavevector $\mathbf{Q}$ remains orthogonal to the crystal surface, i.e., the sound velocities are equal for both geometries. Taking into account the refractive index of

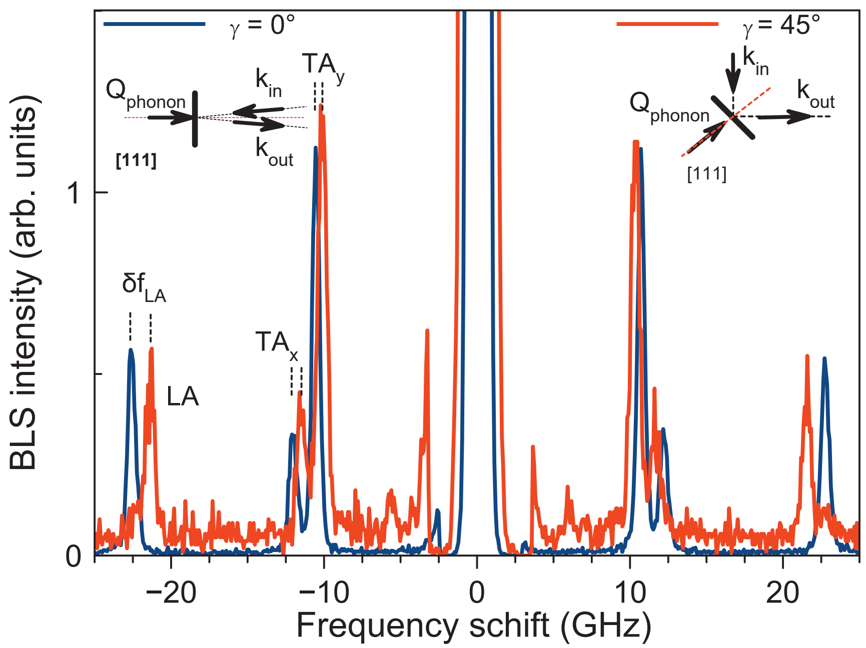

Figure S2: Spectra of Brillouin light scattering (BLS) in back reflection geometry for incidence angles of $\gamma = 0°$ (normal incidence, blue) and $\gamma = 45°$ (red). $T = 5$ K. The insets show schematic presentations of the two experimental geometries. $\mathbf{k_{in}}$ and $\mathbf{k_{out}}$ are the wavevectors of the incident and scattered light, respectively, $\mathbf{Q}$ is the wavevector of the phonon involved in the scattering process, [111] is the crystallographic orientation axis of the sample.

the $Cs_2AgBiBr_6$ perovskite, we can calculate the relative frequency shift of the lines in the two geometries: $\delta f_i(\gamma) = [f_i(\gamma) - f_i(0)]/f_i(0)$ which corresponds to 5.5% for $\gamma = 45°$. Experimentally we observe shifts of the frequencies equal to 5.3%, 4.4%, and 3.8% for the longitudinal and the two transverse acoustical phonons, respectively.

The polarization dependence of the BLS peaks with respect to the crystallographic axes allows us to determine the direction of the c-axis. We observe that the c-axis is randomly oriented along one of three equivalent directions after each cooling cycle from the cubic to the tetragonal crystallographic phase. However, once cooled below T_c, the relative intensities of the BLS peaks remain the same when examining different areas of the sample. This suggests that the direction of the c-axis does not change from one point to another, enabling us to rule out the presence of multiple domains with varying c-axis orientations in the tetragonal phase.

Close to the phase transition temperature, softening of the transverse acoustic modes is observed.

With increasing temperature the acoustic mode frequencies decrease. The softening is particularly strong for the TA_y mode where the BLS shift tends to zero, accompanied by a strong broadening and vanishing of the signal with increasing temperature (see Fig. S3).

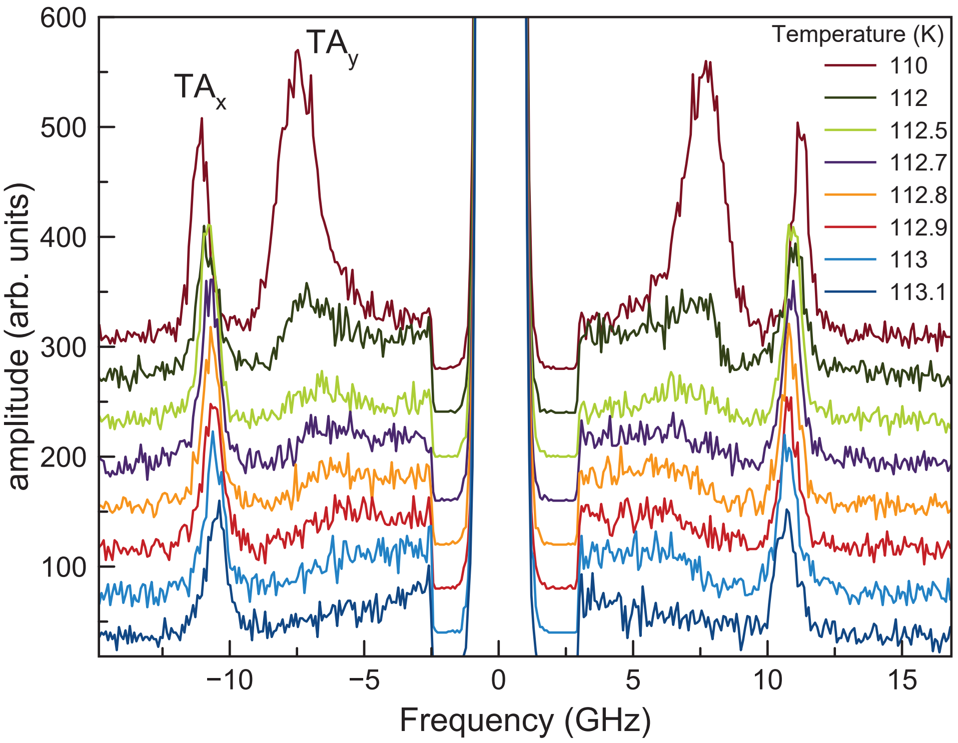

Figure S3: Temperature dependence of the *cw* BLS spectra close to the phase transition.

S3 Theoretical description of cw Brillouin light scattering

The basic equations for the scattering from Ref. (*62*) are reproduced below. These equations become greatly simplified when the refractive index does not depend on the polarization of the light. The experimental data show that the deviation of the refractive index is well below one percent, even in the tetragonal phase, as evidenced from the absence of polarization dependence for the BLS peak positions. We also neglect the dependence of the refractive index on the light frequency.

The following notations are used: the direction of incident light is **q**, the direction of scattered light **q′**. Θ is the angle between **q** and **q′**. Λ and N are wavelength and frequency of the elastic wave. λ and ν are wavelength and frequency of the incident light. Two "main" polarizations for the

incident light are $\mathbf{e}_{1,2}$, and $\mathbf{e}'_{1,2}$ for the scattered light.The indices of refraction are n_μ and $n_{\mu'}$ for $\mathbf{e}_\mu$ and $\mathbf{e}'_\mu$, respectively. The phonon wave vector is $\mathbf{Q}$.

The phonon wavelength and direction are connected to the incident and scattered light by

$$\Lambda_{\mu\mu'} = \frac{\lambda}{\sqrt{n_\mu^2 + n'^2_{\mu'} - 2n_\mu n'_{\mu'} \cos\Theta}} \tag{S1}$$

$$\mathbf{Q}_{\mu\mu'} = \frac{n'_{\mu'}\mathbf{q}' - n_\mu \mathbf{q}}{\left| n'_{\mu'}\mathbf{q}' - n_\mu \mathbf{q} \right|} \tag{S2}$$

The frequency shift is

$$\frac{\delta \nu^s_{\mu\mu'}}{\nu} = \pm \frac{V^s_{\mu\mu'}}{c} \sqrt{n_\mu^2 + n'^2_{\mu'} - 2n_\mu n'_{\mu'} \cos\Theta} \tag{S3}$$

The flux of light scattered by an elementary cubic volume with edge a is given by

$$\Phi^s_{\mu\mu'} = \frac{8\pi^2 kT}{\lambda^4} \frac{n_\mu^4}{(n_\mu^2+1)^2} \frac{n'^4_{\mu'}}{(n'^2_{\mu'}+1)^2} \beta^s_{\mu\mu'} \omega a^3 \epsilon_\mu \tag{S4}$$

where kT gives the temperature in energy units, ω is the scattered light solid angle calculated out of the crystal, ϵ_μ is the illuminance in a given polarization. For systems excluding the monoclinic and triclinic symmetry,

$$\beta^s_{\mu\mu'} = \frac{1}{n_\mu^4} \frac{1}{n'^4_{\mu'}} \frac{\left(n_i^2 n_j^2 e'_{\mu',j} B^s_{ij} e_{\mu,i} \right)^2}{\rho (V^s_{\mu\mu'})^2}\,, \tag{S5}$$

with

$$B^s_{ij} = p'_{ijkl} u^s_k Q_l \tag{S6}$$

where p'_{ijkl} is the tensor of the photoelastic constants. In our case, where the refractive index is isotropic, it is equal to p_{ijkl}: the photoelastic tensor is symmetric with respect to the last two indices (*56*).

S4 Material tensors

The tensor of elastic stiffness is defined (*56*) as

$$\sigma_{ij} = c_{ijkl} \epsilon_{kl}\,, \tag{S7}$$

where σ_{ij} is the stress tensor and ϵ_{kl} is the strain tensor. We use the standard convention in which the symmetric stress and strain tensors are written as six-component vectors associating pairs of tensor indices ij with a single index as $11 \to 1$, $22 \to 2$, $33 \to 3$, $23 \to 4$, $13 \to 5$, $12 \to 6$. With this convention, the rank four symmetric tensor (tensor of elastic stiffness and photoelastic tensor) can be written as a 6×6 matrix.

The photoelastic (opto-elastic) tensor is defined (*56*) through the linear dependence of the components of the dielectric impermeabilities (inverted dielectric tensor) on the components of the strain tensor:

$$\delta \left\{ \varepsilon^{-1} \right\}_{ij} = p_{ijkl} \epsilon_{kl} \, , \tag{S8}$$

where the p_{ijkl} are the components of the photoelastic tensor. For an isotropic dielectric constant $\varepsilon_{ij} = \varepsilon \delta_{ij}$, the change of the dielectric constant is proportional to the same tensor:

$$\delta \varepsilon_{ij} = -\varepsilon^2 p_{ijkl} \epsilon_{kl} \, . \tag{S9}$$

In the cubic phase, the space group of the crystal is #225 and the elastic stiffness tensor and photoelastic tensor have the same form:

$$C_{ij} = \begin{pmatrix} c_{11} & c_{12} & c_{12} & 0 & 0 & 0 \\ c_{12} & c_{11} & c_{12} & 0 & 0 & 0 \\ c_{12} & c_{12} & c_{11} & 0 & 0 & 0 \\ 0 & 0 & 0 & c_{44} & 0 & 0 \\ 0 & 0 & 0 & 0 & c_{44} & 0 \\ 0 & 0 & 0 & 0 & 0 & c_{44} \end{pmatrix}, \quad p_{ij} = \begin{pmatrix} p_{11} & p_{12} & p_{12} & 0 & 0 & 0 \\ p_{12} & p_{11} & p_{12} & 0 & 0 & 0 \\ p_{12} & p_{12} & p_{11} & 0 & 0 & 0 \\ 0 & 0 & 0 & p_{44} & 0 & 0 \\ 0 & 0 & 0 & 0 & p_{44} & 0 \\ 0 & 0 & 0 & 0 & 0 & p_{44} \end{pmatrix} . \tag{S10}$$

Note that in contrast to the main text, in this section and below we use the standard convention and write these four-rank tensors as 6×6 matrices.

Tetragonal phase has the space group #87, class $4/m$, and the tensors are different. Both may

be found in (*56*), § 1.1.4.10.6.5.

$$C_{ij} = \begin{pmatrix} c_{11} & c_{12} & c_{13} & 0 & 0 & c_{16} \\ c_{12} & c_{11} & c_{13} & 0 & 0 & -c_{16} \\ c_{13} & c_{13} & c_{33} & 0 & 0 & 0 \\ 0 & 0 & 0 & c_{44} & 0 & 0 \\ 0 & 0 & 0 & 0 & c_{44} & 0 \\ c_{16} & -c_{16} & 0 & 0 & 0 & c_{66} \end{pmatrix}, \quad p_{ij} = \begin{pmatrix} p_{11} & p_{12} & p_{13} & 0 & 0 & p_{16} \\ p_{12} & p_{11} & p_{13} & 0 & 0 & -p_{16} \\ p_{31} & p_{31} & p_{33} & 0 & 0 & 0 \\ 0 & 0 & 0 & p_{44} & p_{45} & 0 \\ 0 & 0 & 0 & -p_{45} & p_{44} & 0 \\ p_{61} & -p_{61} & 0 & 0 & 0 & p_{66} \end{pmatrix}. \tag{S11}$$

It is worth to mention that, as explained in Refs. (*60, 61*), a change of the dielectric constant in low symmetry crystals is not fully captured by the tensor p_{ij}, but contains also an antisymmetric part which describes the change of the refraction index by twist stress. However, as explained in (*62*), these terms are zero if the refractive index is isotropic. According to the experimental data, the anisotropy of the refractive index in our case may be neglected and the photoelastic interaction may be described by the tensor (S11).

To relate the cubic phase components to the tetragonal ones, an additional step is needed. With the standard crystallographic basis choice, the (normalized) basis vectors in the tetragonal phase $\mathbf{e}^{t}_{j}$ are related to the basis vectors in the cubic phase $\mathbf{e}^{c}_{j}$ by $\mathbf{e}^{t}_{1} = (\mathbf{e}^{c}_{1} - \mathbf{e}^{c}_{2})/\sqrt{2}$, $\mathbf{e}^{t}_{2} = (\mathbf{e}^{c}_{1} + \mathbf{e}^{c}_{2})/\sqrt{2}$, $\mathbf{e}^{t}_{3} = \mathbf{e}^{c}_{3}$. From comparison of the rotated cubic tensor of elastic stiffness with the tetragonal one, one can conclude that the deviation of the tetragonal stiffness from that of the cubic material with parameters c^{c}_{ij} (where $c^{c}_{11} = c_{33}$, $c^{c}_{12} = c_{13}$, and $c^{c}_{44} = c_{44}$) can be expressed via

$$\delta c_{66} = c_{66} - \frac{c_{33} - c_{13}}{2}, \quad \delta c_{11} = c_{11} - \left(c_{44} + \frac{c_{33} + c_{13}}{2}\right), \quad \delta c_{12} = c_{12} + \left(c_{44} - \frac{c_{33} + c_{13}}{2}\right), \quad c_{16}. \tag{S12}$$

Note also that the β-phase of lead halide perovskites has a slightly higher symmetry (space group #140, class $4/mmm$), where $p_{16} = p_{61} = p_{45} = 0$.

Since the elastic stiffness tensor is not known for this material, we performed density functional theory (DFT) calculations to estimate the mode mixing in a bulk material. The DFT calculations were done using the WIEN2k package (*73*). For calculation of the elastic tensor components we use the IRelast package (*74*). First, the structure is optimized using the PBEsol exchange-correlation functional (the optimized cubic lattice constant is 11.19 Å and in the tetragonal phase the lattice constants are $a = 7.84$ Å, $c = 11.32$ Å). Next, for a few selected deformations, the energy as function

of deformation amplitude is calculated and from the fit of the calculated energy the combinations of the elastic tensor components are extracted. The procedure gives the following set of constants in the cubic phase (constants are in GPa):

$$c_{11} = 45.7\,,\ \ c_{12} = 17.2\,,\ \ c_{44} = 7.0\,, \tag{S13}$$

and in the tetragonal phase:

$$\begin{aligned} c_{11} = 41.0,\ \ c_{33} = 42.7, \\ c_{44} = 7.10,\ \ c_{66} = 12.5, \\ c_{12} = 26.2,\ \ c_{13} = 22.5,\ \ c_{16} = -0.5. \end{aligned} \tag{S14}$$

S5 Phonon modes in tetragonal phase

For qualitative analysis it is practical to write the phonon dispersion equation in the tetragonal phase in the coordinate system aligned with the $\mathbf{z}'$ direction. In the coordinates $x'y'z'$ it reads as

$$\left\{C_{ijkl}Q_jQ_k\right\}_{x'y'z'} = \frac{1}{3}\begin{pmatrix} c_T + 2\delta c_{66} & -\frac{2}{\sqrt{3}}c_{16} & -\frac{4}{\sqrt{6}}c_{16} \\ -\frac{2}{\sqrt{3}}c_{16} & c_T + \frac{2}{3}\delta c_{11} & \frac{2\sqrt{2}}{3}\delta c_{11} \\ -\frac{4}{\sqrt{6}}c_{16} & \frac{2\sqrt{2}}{3}\delta c_{11} & c_L + \frac{4}{3}\delta c_{11} \end{pmatrix}. \tag{S15}$$

where

$$\begin{aligned} c_T &= c_{33} - c_{13} + c_{44}\,, \\ c_L &= c_{33} + 2c_{13} + 4c_{44}\,. \end{aligned}$$

In the cubic limit, the dispersion equation gives two degenerate transverse phonon modes $V_{\mathrm{TA}1} = V_{\mathrm{TA}2} \propto c_{33} - c_{13} + c_{44}$ and one longitudinal mode $\propto c_{33} + 2c_{13} + 4c_{44}$, as expected for cubic material. Below we fix the notation as follows: The phonon mode TA_1 is the mode polarized along $\mathbf{x}'$ (normal to the c axis) in the cubic limit, TA_2 is the mode polarized along $\mathbf{y}'$ (non-zero projection on the c axis).

There are three effects of the tetragonal anisotropy on the polarization vectors $\mathbf{U}^s$: (1) there is an admixture of the LA mode to the TA_2 mode, that is proportional to δc_{11}, (2) there is an admixture of the LA mode to the TA_1 mode that is proportional to c_{16}, and (3) there is a mixing of the TA_1

and TA_2 modes, also proportional to c_{16}. Note that each of these admixtures reflects both in the velocities and the displacement vectors $\mathbf{U}^s$. An admixture of LA phonons means that the TA modes are no longer purely transverse, but have a component of the displacement vector along $\mathbf{Q}$. Mixing of the transverse phonons means that the main axes of the transverse phonons is rotated with respect to $\mathbf{x}'$ and $\mathbf{y}'$ in the plane $x'y'$. Exact calculations with the stiffness tensor components extracted from DFT allows us to estimate the mixing of the different modes and shows that the effect of the elastic anisotropy may be calculated perturbatively.

An approximate solution of Eq. (S15) can be written explicitly taking into account that $\delta c_{11} \ll c_L - c_T$ and $c_{16} \ll \delta c_{66} - \delta c_{11}$. For our material this assumption works pretty well.

$$\mathbf{U}^{T1} \approx C_n^{T1} \begin{pmatrix} 1 \\ \sqrt{3}\frac{c_{16}}{\delta c_{66}-\delta c_{11}} \\ 4\sqrt{3}\frac{c_{16}}{c_T-c_L} \end{pmatrix}, \quad \mathbf{U}^{T2} \approx C_n^{T2} \begin{pmatrix} \sqrt{3}\frac{c_{16}}{\delta c_{66}-\delta c_{11}} \\ 1 \\ \frac{2\sqrt{2}}{3}\frac{\delta c_{11}}{c_L-c_T} \end{pmatrix}, \quad \mathbf{U}^{L} \approx C_n^{L} \begin{pmatrix} \frac{4}{\sqrt{3}}\frac{c_{16}}{c_L-c_T} \\ \frac{2\sqrt{2}}{3}\frac{\delta c_{11}}{c_L-c_T} \\ 1 \end{pmatrix}, \tag{S16}$$

where the C_n^s are the normalization coefficients.

From Eq. (S16) one may qualitatively understand the behavior of the energies and displacement vectors. The "zeroth order" modes contain squares of velocities proportional to $c_T + 2/3\ \delta c_{66}$, $c_T + 2/3\ \delta c_{11}$ and c_L with their displacement vectors aligned as follows: TA_1 along $\mathbf{x}'$, TA_2 along $\mathbf{y}'$, and LA along $\mathbf{z}'$. Then, in first order, the displacement vectors are rotated in the $x'z'$ plane proportional to c_{16}, in the $x'y'$ plane also proportional to c_{16}, and in the $y'z'$ plane proportional to δc_{11}.

The phonon amplitudes $\mathbf{U}^s$ may be recalculated to strain tensor components:

$$\epsilon_{ij}^s = \frac{1}{2}(U_i^s Q_j + U_j^s Q_i) . \tag{S17}$$

In the coordinate system $x'y'z'$, only $Q_{z'}$ is non-zero. As a result, only three components of the strain tensor are non-zero, namely

$$\epsilon_{z'z'}^s = U_{z'}^s , \quad \epsilon_{y'z'}^s = \frac{1}{2}U_{y'}^s , \quad \epsilon_{z'x'}^s = \frac{1}{2}U_{x'}^s . \tag{S18}$$

In our case, the modes of interest are the LA phonons with the dominatig component of the strain tensor $\epsilon_{z'z'}$ and the TA_2 phonons with the dominating component $\epsilon_{y'z'}$

S6 Generation of strain wave

Now let us discuss a more formal solution of the problem. The wave equation with the right part reads

$$\rho \frac{d^2 u_i}{dt^2} = \frac{\partial \sigma_{ij}}{\partial x_j}\,, \tag{S19a}$$

$$\sigma_{ij} = c_{ijkl}\epsilon_{kl} + \Sigma_{ij}\,, \tag{S19b}$$

$$\epsilon_{kl} = \frac{1}{2}\left(\frac{\partial u_k}{\partial x_l} + \frac{\partial u_l}{\partial x_k}\right)\,. \tag{S19c}$$

Here Σ_{kl} is the stress induced by the laser light, ϵ is the deformation tensor, and $\mathbf{u}$ is the displacement vector.

Equation (1) is the Fourier transform of (S19) without the right part. The Fourier transform of the original problem with the right part included gives

$$\left(\rho\omega^2\delta_{il} - c_{ijkl}k_j k_k\right) U_l(\mathbf{k},\omega) = \Phi_i(\mathbf{k},\omega)\,, \tag{S20}$$

with the right part Fourier transform of the driving force $\mathbf{\Phi}$:

$$\Phi_i(\mathbf{k},\omega) = \int \Phi_i(\mathbf{r},t) e^{-i(\mathbf{k}\cdot\mathbf{r}-\omega t)} d\mathbf{r}dt.$$

Without loss of generality, below we give results only for the photostriction contribution (5). Direct calculations show that for the pump pulse at the surface with normal $\mathbf{n}$

$$\Phi_i(\mathbf{k},\omega) = -n_i\alpha_i\rho_{X,S}\left[2\pi\delta(\omega) - \frac{2i}{\omega}\right]\frac{2n_x n_y n_z \zeta}{(n_x^2+k_x^2)(n_y^2+k_y^2)(n_z^2+k_z^2)}. \tag{S21}$$

The solution of (S20) gives three waves propagating with velocities corresponding to acoustic eigenmodes, and the amplitude of the mode s is proportional to $\mathbf{\Phi}\cdot\mathbf{U}^{\mathrm{s}}$.

The displacement field in the wave after the pulse is proportional to

$$u_i(\mathbf{r},t) \sim \sum_{s=\mathrm{LA},\mathrm{TA}_x,\mathrm{TA}_y} A_s U_i^s f\left(t - \frac{\mathbf{r}\cdot\mathbf{n}}{V_s}\right)\,, \tag{S22}$$

where f is the envelope of the strain pulse. The strain tensor is

$$\epsilon_{ij}(\mathbf{r},t) \sim -\sum_{s=\mathrm{LA},\mathrm{TA}_x,\mathrm{TA}_y} \frac{A_s}{2V_s} f'\left(t - \frac{\mathbf{r}\cdot\mathbf{n}}{V_s}\right)\left(U_i^s n_j + U_j^s n_i\right)\,. \tag{S23}$$

Note that the strain tensor for different modes is proportional not only to the amplitude $A_s \sim \mathbf{\Phi}\cdot\mathbf{U}^s$ but also to $\epsilon_{ij}^s = \frac{1}{2}(U_i^s Q_j + U_i^s Q_i)$. The polarization dependence of the signal is defined only by ϵ_{ij}^s and the components of the photoelastic tensor, see the details in the next section S7.

S7 Pump-probe Brillouin signal

In the pump-probe experiment, the measured signal is the interference between the probe pulse reflected from the surface and the probe pulse reflected from the coherent phonons generated by the pump pulse. The amplitude of the first is $R\mathbf{E_0}$ where the reflection coefficient is $R = \frac{1-n}{1+n}$. It can be demonstrated that the electric field of the scattered light can be written (see e.g. Refs. (*24, 59*)) in first order as

$$\mathbf{E}(z) \approx \mathbf{E}_0(z) + k^2 \int_{-\infty}^{\infty} G(z, \tilde{z}) \delta\hat{\varepsilon}(\tilde{z}) \mathbf{E}_0(\tilde{z}) \mathrm{d}\tilde{z}, \tag{S24}$$

where $\delta\hat{\varepsilon}$ is the variation of the dielectric constant proportional to the strain (S9) and $G(z, z')$ is the Green function of the wave equation

$$\left[\frac{\partial^2}{\partial z^2} + k^2\right] G(z, \tilde{z}) = \delta(z - \tilde{z})\,. \tag{S25}$$

In an infinite 1D system, the Green function is given by

$$G(z, \tilde{z}) = -\frac{i}{2k} e^{ik|z-\tilde{z}|}\,, \tag{S26}$$

and is independent of the polarization of light. The dielectric constant change is proportional (S9) to the strain in the pulse (S23):

$$\delta\varepsilon_{ij}(z, t) \sim \sum_{s=\mathrm{LA},\mathrm{TA}_x,\mathrm{TA}_y} \frac{\varepsilon^2 A_s}{V_s} f'\left(t - \frac{z}{V_s}\right) B^s_{ij}\,, \tag{S27}$$

where the symmetry of the photoelastic tensor is used to rewrite the result via the matrices defined in Sec. S3 to describe the *cw*-Brillouin scattering. Let us postpone the discussion of the polarization dependence of the signal and concentrate on its amplitude first. Since the acoustic phonons are generated by a short laser pulse, it is natural to assume that the profile of the generated pulse is well localized. Let us assume that the reflection coefficient given by the pulse envelope f' is r (i.e., for $\delta\varepsilon(z, t) \sim \frac{\varepsilon^2}{V} f'\left(t - \frac{z}{V}\right)$ the solution of the wave equation is given by $\mathbf{E}(z) = \mathbf{A}_0 e^{ikz} + r\mathbf{A}_0 e^{-ikz}$). Then, the reflection coefficient is given by

$$R_{ij} = -\frac{1-n}{1+n}\delta_{ij} + \sum_{s=\mathrm{LA},\mathrm{TA}_x,\mathrm{TA}_y} A_s r e^{2ikV_s t} B^s_{ij}\,. \tag{S28}$$

This results in signals oscillating at the frequencies kV_s with amplitude

$$2rA_s \sum_{ij} e_i B^s_{ij} e_j \tag{S29}$$

where the e_i are the components of the incident light polarization vector. Note that the polarization dependence of the signal amplitude is up to the square equal to the amplitude of the *cw*-Brillouin signal in co-polarization, c.f. Eq. (S5). A comparison of the obtained experimental results can be found in Fig. S4.

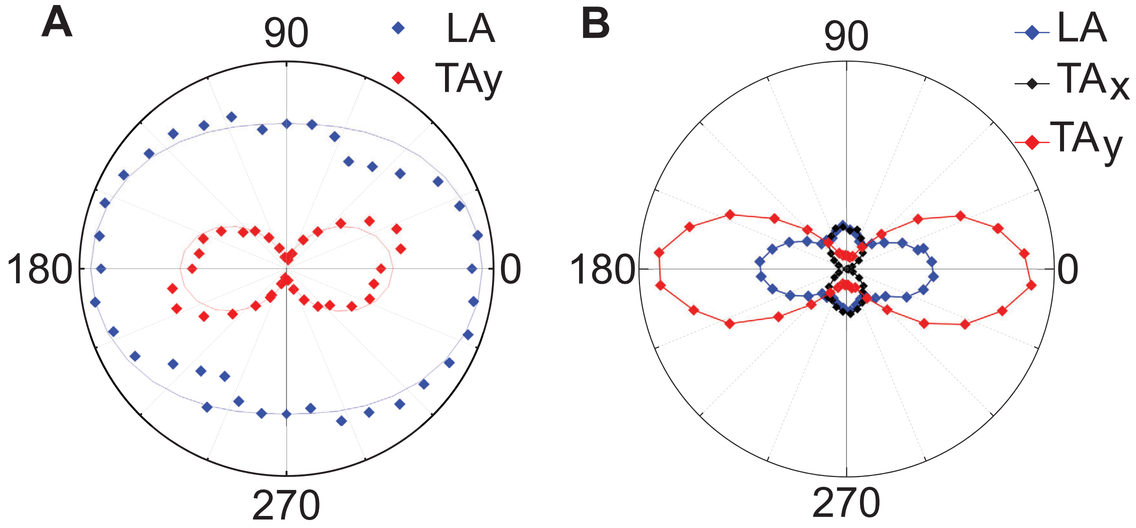

Figure S4: **Comparison of the signal amplitude** obtained in the LF pump-probe measurements (A) and the *cw* BLS measurements (B). The pump-probe measurements were done at $h\nu_{\text{pump}} = 2.818$ eV and $h\nu_{\text{probe}} = 2$ eV. The *cw* BLS was measured in back reflection geometry in co-polarization of the incident and scattered light at the photon energy $h\nu_{\text{BLS}} = 2.287$ eV for $T = 5$ K.

To simplify the analysis of the polarization dependence of the signal, it is constructive to rewrite the photoelastic tensor in the coordinates $x'y'z'$. Then, the change of the components of the dielectric tensor is proportional to its 3rd column for the LA phonons ($\epsilon_{z'z'}$), to the 4th column for the TA_2 phonons ($\epsilon_{y'z'}$), and to the 5th column for the TA_1 phonons ($\epsilon_{x'z'}$). Since the light propagates along the $\mathbf{z}'$ direction, for the calculation of the reflected light we need only the following components of the dielectric tensor: $\delta\varepsilon_{x'x'}$, $\delta\varepsilon_{y'y'}$, $\delta\varepsilon_{x'y'}$. The result for a tetragonal $4/m$ crystal is the following:

$$\delta\varepsilon^{LA}_{x'x'} \propto 2p_{12} + p_{13}\,, \tag{S30a}$$

$$\delta\varepsilon^{LA}_{y'y'} \propto (2p_{11} + 2p_{33} + p_{13} + 4p_{31} - 8p_{44})/3\,, \tag{S30b}$$

$$\delta\varepsilon^{LA}_{x'y'} \propto 2(p_{45} - p_{61})/\sqrt{3}\,, \tag{S30c}$$

$$\delta\varepsilon^{TA_2}_{x'x'} \propto p_{12} - p_{13}\,, \tag{S31a}$$

$$\delta\varepsilon^{TA_2}_{y'y'} \propto (p_{11} - 2p_{33} - p_{13} + 2p_{31} + 2p_{44})/3\,, \tag{S31b}$$

$$\delta\varepsilon^{TA_2}_{x'y'} \propto -(p_{45} + p_{16})/\sqrt{3}\,, \tag{S31c}$$

$$\delta\varepsilon^{TA_1}_{x'x'} \propto p_{16}\,, \tag{S32a}$$

$$\delta\varepsilon^{TA_1}_{y'y'} \propto -(2p_{45} + p_{61})/3\,, \tag{S32b}$$

$$\delta\varepsilon^{TA_1}_{x'y'} \propto (p_{66} - p_{44})/\sqrt{3}\,. \tag{S32c}$$

Let us discuss qualitatively the results in Eqs. (S30-S32). We consider the sequence: 1. isotropic case → 2. cubic $m\bar{3}m$ → 3. tetragonal $4/mmm$ → 4. tetragonal $4/m$.

1. In the fully isotropic case there are two independent components p_{11} and p_{12}, while all others $p_{11} = p_{33}$, $p_{12} = p_{13} = p_{31}$, $p_{44} = p_{66} = \frac{p_{11}-p_{12}}{2}$, $p_{45} = p_{16} = p_{61} = 0$ which leads to a polarization-independent reflection only for the LA phonons, while no signal from TA phonons can be detected even if they were generated.

2. In the cubic symmetry, there is an additional non-zero component $p^c_c = p^c_{11} - p^c_{12} - 2p^c_{44}$ and Eqs. (S30-S32) reduce to:

$$\delta\varepsilon^{LA}_{x'x'} = \delta\varepsilon^{LA}_{y'y'} \propto 3p_{12} + p^c_c\,, \tag{S33a}$$

$$\delta\varepsilon^{TA_2}_{x'x'} = -\delta\varepsilon^{TA_2}_{y'y'} \propto p^c_c\,, \tag{S33b}$$

$$\delta\varepsilon^{TA_1}_{x'y'} = \delta\varepsilon^{TA_1}_{y'x'} \propto p^c_c\,. \tag{S33c}$$

Eqs. (S33) show that the signal for the LA phonons is still isotropic, and the TA phonons give a polarization dependent four-lobe signal with the maximum/minimum along the x', y' axes for the TA_2/TA_1 phonons (i.e., the signal is proportional to $|\cos 2\phi|$, $|\sin 2\phi|$, respectively). Note that the amplitude of reflection for both TA phonons is given by the same constant p_c.

3. In the $4/mmm$ system, there are 7 independent photoelastic tensor components, only $p_{45} = p_{16} = p_{61} = 0$. In this case, the TA_1 signal is expected to be the same as in the cubic case. To explicitly show the difference from the cubic symmetry, we write the results for the LA and TA_2 phonons as:

$$\delta\varepsilon_{x'x'}^{LA} \propto 3p_{12} + p_c + 3(p_{13} - p_{12}) \,, \tag{S34a}$$

$$\delta\varepsilon_{y'y'}^{LA} \propto 3p_{12} + p_c + \frac{2}{3}(p_{33} - p_{11}) + \frac{1}{3}(p_{13} - p_{12}) + \frac{4}{3}(p_{31} - p_{12}) + \frac{8}{3}(p_{66} - p_{44}) \,, \tag{S34b}$$

and

$$\delta\varepsilon_{x'x'}^{TA_2} \propto p_c - 2(p_{13} - p_{12}) \,, \tag{S35a}$$

$$\delta\varepsilon_{y'y'}^{TA_2} \propto -p_c - \frac{1}{3}(p_{33} - p_{11}) - \frac{2}{3}(p_{13} - p_{12}) + \frac{4}{3}(p_{31} - p_{12}) - \frac{4}{3}(p_{66} - p_{44}) \,, \tag{S35b}$$

In this case, the signal can have a rather complex shape. We note that the signal for the TA_2 phonons is linearly polarized if the crystal is almost isotropic normal to the [001] axis which means that $\delta\varepsilon_{x'x'}^{TA_2} \approx 0$, while $\delta\varepsilon_{y'y'}^{TA_2}$ is large. A realistic scenario is the combination of the photoelastic tensor being isotropic in the $x'y'$ plane (which leads to $p_c = 0$) and the same effect of strain along and normal to the c axis on the dielectric constant (which means that $p_{12} \approx p_{13}$).

4. In the $4/m$ system, there are 10 independent photoelastic tensor components, in addition to the $4/mmm$ case, there are the non-zero p_{45}, p_{16} and $p_{61} \neq 0$ components. In comparison to $4/m$, their main effect is rotation of the signal with respect to the crystallographic axes.

S8 Thermoelastic mechanism of strain generation

As explained in the main text, there are two mechanisms of strain pulse generation caused by the femtosecond pulses: the first one is due to the lattice temperature increase (4) and the second one is due to the photostriction (5). Both result in shear strain as explained in the main text. Below we estimate the amplitude of the phonons generated by the first mechanism and demonstrate that for our experimental conditions, a strong dependence of the generated strain pulse amplitude on the temperature would be expected.

Let us concentrate on the case of low frequency (LF) laser repetiton rate. The pulses have the photon energy $h\nu_{\text{pump}} = 2.818$, ($\lambda_{\text{pump}} = 440$ nm). The average laser power of 4 μW (for completeness, the probe pulse power is 80 μW at $\lambda_{\text{probe}} = 620$ nm) is concentrated in short (~ 150 fs) laser pulses with the repetition rate 30 kHz. So, each pulse has the energy $\sim 1.33 \cdot 10^{-10}$ J. This

energy is assumed to be uniformly distributed in the laser spot with diameter 5 μm, which leads to the fluence $\Psi_{\text{pump}} = 0.65 \cdot 10^{-3}$ J/cm^2 of the incident pump pulse.

The energy of the incident light leads to an almost immediate increase of the lattice temperature which we intend to estimate and to the generation of excitons with energy $E_g \sim 2$ eV. Then, the total energy in the pulse which contributes to heating of the lattice can be estimated as $\Psi_{\delta T} = (1 - R)\frac{h\nu_{\text{pump}} - E_g}{h\nu_{\text{pump}}}\Psi_{\text{pump}}$. For $\lambda_{\text{pump}} = 440$ nm, the refractive index is $n_{\text{pump}} = 2.406$ (*49*) and $R_E = |(n-1)/(n+1)|^2 = 0.17$, which results in $\Psi_{\delta T} = 1.6 \cdot 10^{-4}$ J/cm^2.

Now let us take into account that the energy of the pump pulse is distributed in the volume of the sample. The energy density is proportional to $P_{\delta T}(z') = P^0_{\delta T} e^{-\alpha z'}$ where α is the absorption coefficient. For $\lambda_{\text{pump}} = 440$ nm, the absorption coefficient is $\alpha = 1.53 \cdot 10^5$ cm^{-1} (*49*). From $\Psi_{\delta T} = \int_0^\infty P_{\delta T}(z')\mathrm{d}z'$ it follows that $P^0_{\delta T} = \Psi_{\delta T}\alpha = 24.5$ J/cm^3

Next, the energy transferred to the lattice can be recalculated into an increase of the temperature. Let us concentrate on the temperature increase at the surface. As shown in Ref. (*34*), the heat capacity below the phase transition is perfectly well described by the Debye model. We recalculate the heat capacity extracted from experiment to the volume (the mass density is $\rho = 5$ g/cm^3) and use it to compute the temperature increase. In the Debye model, the energy of the lattice (neglecting optical phonon modes) is (*80*)

$$E_D(T) = E_0 + 3Nk_0 \cdot T D_3\left(\frac{T_D}{T}\right), \quad D_3(t) = \frac{3^3}{t}\int_0^t \frac{x^3}{e^x - 1}\mathrm{d}x\,. \tag{S36}$$

where $3Nk_0$ and T_D are taken from experiment (*34*).

The surface temperature increase δT_0 can then be found from the equation

$$E_D(T + \delta T_0) = E_D(T) + P^0_{\delta T}\,. \tag{S37}$$

Equation (S37) can be solved numerically. From $\lim_{T\to 0} E_D(T) \sim T^3$, it follows that the same energy leads to a different increase of the temperature for different temperatures of the lattice before the pulse.

For instance, $P^0_{\delta T} = 24.5$ J/cm^3 at the temperature 5 K leads to $\delta T(5\text{ K}) = 46.7$ K, while for the temperature 50 K it gives $\delta T(50\text{ K}) = 24.6$ K and for the temperature 100 K $\delta T(100\text{ K}) = 22.6$ K. The change of temperature for a given pulse energy as function of the initial sample temperature is shown in Figure S5. For comparison we show the change of temperature also for smaller pulse

energies, the change of temperature e.g. for $P^0_{\delta T} = 5$ J/cm^3 in the given by $\delta T(T = 5\text{ K}) = 21.4$ K, while it is $\delta T(T = 80\text{ K}) = 4.6$ K, demonstrating a difference by almost a factor 5.

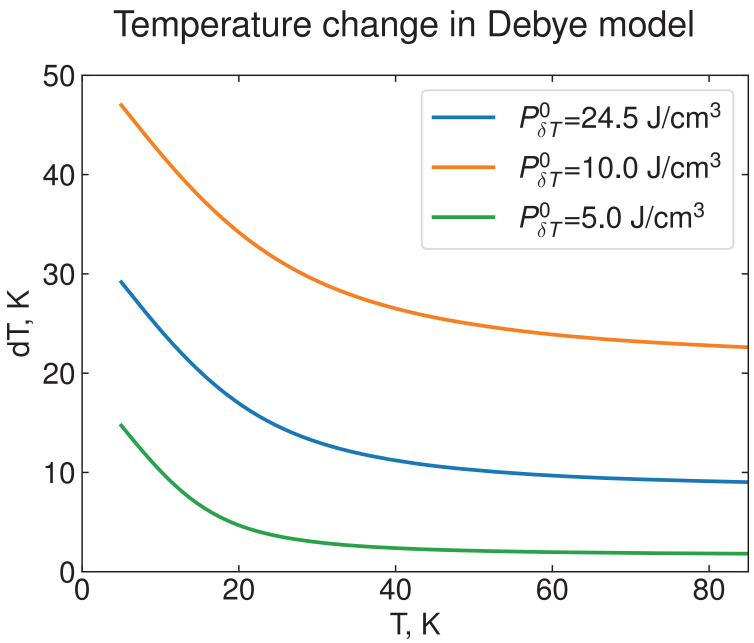

Figure S5: Change of temperature due to heating calculated after (S37). The parameters of the Debye model ($T_D = 114$ K, $C_V(T = \infty) = 1.2$ J/cm^3) are extracted from Ref. (*34*).

Next, the heating may be recalculated to the strain amplitude. From Fig. 1b of Ref. (*34*) it follows that $\beta_c \approx -9 \cdot 10^{-5}$ K^{-1} and $\beta_{a,b} \approx 7 \cdot 10^{-5}$ K^{-1}. As a result, for the temperature mechanism (4), the amplitude of the strain field is of the order $\epsilon \sim \beta_i \delta T$, which can be estimated to be about 10^{-3}. Note that $\delta T(5\text{ K})/\delta T(50\text{ K}) \sim 2$ means that the pure temperature effect of strain generation should result in a strong (at least two times) change of the signal amplitude when the temperature of the sample changes from 5 to 30 K. The insensitivity of the signal with respect to temperature in this range indirectly confirms that the main effect is not related to the temperature mechanism (4), but due to the photostriction (5).

A similar analysis may be applied to estimate the effect of the phorostriction mechanism (5). Now, we consider the exciton population which corresponds to the density of absorbed photons in pump pulse $(1 - R)\Psi_{\text{pump}}/h\nu_{\text{pump}} \approx 1.3 \cdot 10^{14}$ 1/cm^2. As a result, the exciton density near the surface may be estimated as $\rho_X(0) \approx 2.0 \cdot 10^{20}$ 1/cm^3. As long as we assume that the photostriction mechanism is at least as strong as the temperature mechanism, we may estimate the lower bound

for the photostriction coefficient as $\alpha_i \sim 5.0 \cdot 10^{-24}$ cm^3. In Ref. (*39*), the photostriction coefficient for $CsPbI_3$ is estimated to be $0.5\%/(e/f.u.)$ which, taking into account that $V_{f.u.} \approx 250$ Å^3, is approximately $4 \cdot 10^{-23}$ cm^3.

For the used high frequency repetition rate (HF), the pump laser power is 500 μW at ($\lambda^{\mathrm{HF}}_{\mathrm{pump}} =$ 470 nm and probe is 200 μW at $\lambda^{\mathrm{HF}}_{\mathrm{probe}} = 530$ nm, which, noting that at this wavelength $n = 2.36$ and $\alpha = 3.13 \cdot 10^4$ cm^{-1}, results in $P^0_{\delta T} = 0.04$ J/cm^3. This may be recomputed to heatings of $\delta T^{\mathrm{HF}}(5\ \mathrm{K}) = 2.5$ K and $\delta T^{\mathrm{HF}}(50\ \mathrm{K}) = 0.04$ K.

S9 Generation of strain pulse: Dependence on wavelength and polarization of the exciting optical pulse

Data of pump-probe signal amplitude as function of pump wavelength obtained from the HF 80 MHz laser setup using a probe wavelength of 530 nm are shown in Fig. S6A. FFT spectra measured for two different polarizations (∘ and ×) of the pump beam are shown in Fig. S6B. Here, ∘ and × correspond to linear polarizations which are parallel and orthogonal to the projection of the *c*-axis on the surface plane, respectively. It follows that there is no the dependence of the signal amplitude on the polarization of the pump beam. Therefore, we exclude a contribution from impulsive stimulated Brillouin scattering via the electrostriction mechanism as source of the coherent phonon generation in $Cs_2AgBiBr_6$, which would show a pronounced polarization variation.

The proposed photostriction mechanism is attributed to photogenerated carriers or excitons due to direct absorption of the pump beam, which is independent of the polarization of the exciting light. Note, that there is no anisotropy of refractive index in our particular crystal as confirmed by *cw* BLS measurements, mentioned in section S3). The importance of photogenerated carriers is supported by the absence of a pump-probe signal when the excitation energy is below the direct band gap (see Fig. S6A). Whether the photostrictive mechanism in $Cs_2AgBiBr_6$ is of impulsive or displacive nature remains an open question. In general, the duration of the stress created by photoexcited carriers can be shorter than the period of a phonon oscillation because fast relaxation due to polaron formation was reported in $Cs_2AgBiBr_6$ (*32, 35*). This point requires further studies.

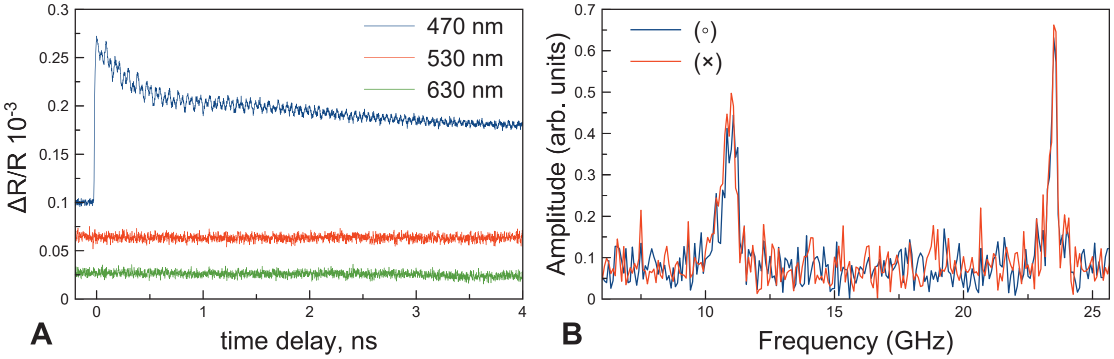

Figure S6: **Dependence on wavelength and polarization of pump pulse. A.** Signal amplitude as a function of pump wavelength at T = 5 K. The probe wavelength is 530 nm. **B.** FFT spectra of the oscillatory signals due to coherent phonons obtained for pump polarization parallel (○) and perpendicular (×) to the projection of the *c*-axis on the surface at T = 5 K. The polarization of the probe is along the projection of the *c*-axis on the surface.

REFERENCES AND NOTES

1. I. Carraro-Haddad, D. L. Chafatinos, A. S. Kuznetsov, I. A. Papuccio-Fernández, A. A. Reynoso, A. Bruchhausen, K. Biermann, P. V. Santos, G. Usaj, A. Fainstein, Solid-state continuous time crystal in a polariton condensate with a built-in mechanical clock. *Science* **384**, 995–1000 (2024).

2. C. Brüggemann, A. V. Akimov, A. V. Scherbakov, M. Bombeck, C. Schneider, S. Höfling, A. Forchel, D. R. Yakovlev, M. Bayer, Laser mode feeding by shaking quantum dots in a planar microcavity. *Nat. Photonics* **6**, 30–34 (2012).

3. O. Ortiz, P. Priya, A. Rodriguez, A. Lemaitre, M. Esmann, N. D. Lanzillotti-Kimura, Topological optical and phononic interface mode by simultaneous band inversion. *Optica* **8**, 598–605 (2021).

4. G.-W. Chern, K.-H. Lin, C.-K. Sun, Transmission of light through quantum heterostructures modulated by coherent acoustic phonons. *J. Appl. Phys.* **95**, 1114–1121 (2004).

5. O. Matsuda, O. B. Wright, Reflection and transmission of light in multilayers perturbed by picosecond strain pulse propagation. *J. Opt. Soc. Am. B* **19**, 3028–3041 (2002).

6. E. Baldini, A. Dominguez, T. Palmieri, O. Cannelli, A. Rubio, P. Ruello, M. Chergui, Exciton control in a room temperature bulk semiconductor with coherent strain pulses. *Sci. Adv.* **5**, eaax2937 (2019).

7. K.-H. Lin, G.-W. Chern, Y.-K. Huang, C.-K. Sun, Terahertz electron distribution modulation in piezoelectric $In_xGa_{1-x}N$/GaN multiple quantum wells using coherent acoustic nanowaves. *Phys. Rev. B* **70**, 073307 (2004).

8. E. S. K. Young, A. V. Akimov, M. Henini, L. Eaves, A. J. Kent, Subterahertz acoustical pumping of electronic charge in a resonant tunneling device. *Phys. Rev. Lett.* **108**, 226601 (2012).

9. R. Gu, V. Juve, C. Laulhe, H. Boutanfif, G. Vaudel, A. Poirier, B. Dkhil, P. Hollander, C. Paillard, M. C. Weber, D. Sando, S. Fusil, V. Garcia, P. Ruello, Temporal and spatial tracking

of ultrafast light-induced strain and polarization modulation in a ferroelectric thin film. *Sci. Adv.* **9**, eadi1160 (2023).

10. A. V. Scherbakov, A. S. Salasyuk, A. V. Akimov, X. Liu, M. Bombeck, C. Brüggemann, D. R. Yakovlev, V. F. Sapega, J. K. Furdyna, M. Bayer, Coherent magnetization precession in ferromagnetic (Ga,Mn) as induced by picosecond acoustic pulses. *Phys. Rev. Lett.* **105**, 117204 (2010).

11. A. von Reppert, L. Willig, J.-E. Pudell, S. P. Zeuschner, G. Sellge, F. Ganss, O. Hellwig, J. A. Arregi, V. Uhlíř, A. Crut, M. Bargheer, Spin stress contribution to the lattice dynamics of FePt. *Sci. Adv.* **6**, eaba1142 (2020).

12. V. V. Temnov, Ultrafast acousto-magneto-plasmonics. *Nat. Photonics* **6**, 728–736 (2012).

13. M. R. Armstrong, E. J. Reed, K. Y. Kim, J. H. Glownia, W. M. Howard, E. L. Piner, J. C. Roberts, Observation of terahertz radiation coherently generated by acoustic waves. *Nat. Phys.* **5**, 285–288 (2009).

14. E. Rongione, O. Gueckstock, M. Mattern, O. Gomonay, H. Meer, C. Schmitt, R. Ramos, T. Kikkawa, M. Mičica, E. Saitoh, J. Sinova, H. Jaffrès, J. Mangeney, S. T. B. Goennenwein, S. Geprägs, T. Kampfrath, M. Kläui, M. Bargheer, T. S. Seifert, S. Dhillon, R. Lebrun, Emission of coherent THz magnons in an antiferromagnetic insulator triggered by ultrafast spin–phonon interactions. *Nat. Commun.* **14**, 1818 (2023).

15. B. Audoin, Principles and advances in ultrafast photoacoustics; applications to imaging cell mechanics and to probing cell nanostructure. *Photoacoustics* **31**, 100496 (2023).

16. C. Thomsen, H. T. Grahn, H. J. Maris, J. Tauc, Surface generation and detection of phonons by picosecond light pulses. *Phys. Rev. B* **34**, 4129–4138 (1986).

17. O. Matsuda, M. C. Larciprete, R. Li Voti, O. B. Wright, Fundamentals of picosecond laser ultrasonics. *Ultrasonics* **56**, 3–20 (2015).

18. G. Tas, H. J. Maris, Electron diffusion in metals studied by picosecond ultrasonics. *Phys. Rev. B* **49**, 15046–15054 (1994).

19. O. B. Wright, Ultrafast nonequilibrium stress generation in gold and silver. *Phys. Rev. B* **49**, 9985–9988 (1994).

20. E. S. K. Young, A. V. Akimov, R. P. Campion, A. J. Kent, V. Gusev, Picosecond strain pulses generated by a supersonically expanding electron-hole plasma in GaAs. *Phys. Rev. B* **86**, 155207 (2012).

21. M. F. Pascual-Winter, A. Fainstein, B. Jusserand, B. Perrin, A. Lemaitre, Spectral responses of phonon optical generation and detection in superlattices. *Phys. Rev. B* **85**, 235443 (2012).

22. V. L. Korenev, M. Salewski, I. A. Akimov, V. F. Sapega, L. Langer, I. V. Kalitukha, J. Debus, R. I. Dzhioev, D. R. Yakovlev, D. Müller, C. Schröder, H. Hövel, G. Karczewski, M. Wiater, T. Wojtowicz, Y. G. Kusrayev, M. Bayer, Long-range p-d exchange interaction in a ferromagnet–semiconductor hybrid structure. *Nat. Phys.* **12**, 85–91 (2016).

23. T. Pezeril, P. Ruello, S. Gougeon, N. Chigarev, D. Mounier, J. M. Breteau, P. Picart, V. Gusev, Generation and detection of plane coherent shear picosecond acoustic pulses by lasers: Experiment and theory. *Phys. Rev. B* **75**, 174307 (2007).

24. O. Matsuda, O. B. Wright, D. H. Hurley, V. Gusev, K. Shimizu, Coherent shear phonon generation and detection with picosecond laser acoustics. *Phys. Rev. B* **77**, 224110 (2008).

25. C. L. Poyser, W. B. York, D. Srikanthreddy, B. A. Glavin, T. L. Linnik, R. P. Campion, A. V. Akimov, A. J. Kent, Phonon spectroscopy with chirped shear and compressive acoustic pulses. *Phys. Rev. Lett.* **119**, 255502 (2017).

26. C.-C. Chen, H.-M. Huang, T.-C. Lu, H.-C. Kuo, C.-K. Sun, Magnitude-tunable sub-THz shear phonons in a non-polar GaN multiple-quantum-well p-i-n diode. *Appl. Phys. Lett.* **100**, 201905 (2012).

27. P. Ruello, V. E. Gusev, Physical mechanisms of coherent acoustic phonons generation by ultrafast laser action. *Ultrasonics* **56**, 21–35 (2015).

28. M. Lejman, G. Vaudel, I. C. Infante, P. Gemeiner, V. E. Gusev, B. Dkhil, P. Ruello, Giant ultrafast photo-induced shear strain in ferroelectric $BiFeO_3$. *Nat. Commun.* **5**, 4301 (2014).

29. P. Guo, Y. Xia, J. Gong, C. C. Stoumpos, K. M. McCall, G. C. B. Alexander, Z. Ma, H. Zhou, D. J. Gosztola, J. B. Ketterson, M. G. Kanatzidis, T. Xu, M. K. Y. Chan, R. D. Schaller, Polar fluctuations in metal halide perovskites uncovered by acoustic phonon anomalies. *ACS Energy Lett.* **2**, 2463–2469 (2017).

30. P.-A. Mante, C. C. Stoumpos, M. G. Kanatzidis, A. Yartsev, Directional negative thermal expansion and large Poisson ratio in $CH_3NH_3PbI_3$ perovskite revealed by strong coherent shear phonon generation. *J. Phys. Chem. Lett.* **9**, 3161–3166 (2018).

31. L. Schmidt-Mende, V. Dyakonov, S. Olthof, F. Ünlü, K. M. T. Lê, S. Mathur, A. D. Karabanov, D. C. Lupascu, L. M. Herz, A. Hinderhofer, F. Schreiber, A. Chernikov, D. A. Egger, O. Shargaieva, C. Cocchi, E. Unger, M. Saliba, M. M. Byranvand, M. Kroll, F. Nehm, K. Leo, A. Redinger, J. Höcker, T. Kirchartz, J. Warby, E. Gutierrez-Partida, D. Neher, M. Stolterfoht, U. Würfel, M. Unmüssig, J. Herterich, C. Baretzky, J. Mohanraj, M. Thelakkat, C. Maheu, W. Jaegermann, T. Mayer, J. Rieger, T. Fauster, D. Niesner, F. Yang, S. Albrecht, T. Riedl, A. Fakharuddin, M. Vasilopoulou, Y. Vaynzof, D. Moia, J. Maier, M. Franckevičius, V. Gulbinas, R. A. Kerner, L. Zhao, B. P. Rand, N. Glück, T. Bein, F. Matteocci, L. A. Castriotta, A. D. Carlo, M. Scheffler, C. Draxl, Roadmap on organic–inorganic hybrid perovskite semiconductors and devices. *APL Materials* **9**, 109202 (2021).

32. B. Wu, Q. Xu, M. Manjappa, M. Feng, S. Ye, J. Fu, S. Lie, T. Yin, F. Wang, T. W. Goh, P. C. Harikesh, Y. K. E. Tay, Z. X. Shen, F. Huang, R. Signh, G. Zhou, F. Gao, T. C. Sum, Strong self-trapping by deformation potential limits photovoltaic performance in bismuth double perovskite. *Sci. Adv.* **7**, eabd3160 (2021).

33. W. Tress, M. T. Sirtl, $Cs_2AgBiBr_6$ double perovskites as lead-free alternatives for perovskite solar cells? *Solar RRL* **6**, 2100770 (2022).

34. L. Schade, A. D. Wright, R. D. Johnson, M. Dollmann, B. Wenger, P. K. Nayak, D. Prabhakaran, L. M. Herz, R. Nicholas, H. J. Snaith, P. G. Radaelli, Structural and optical properties of $Cs_2AgBiBr_6$ double perovskite. *ACS Energy Lett* **4**, 299–305 (2019).

35. S. J. Zelewski, J. M. Urban, A. Surrente, D. K. Maude, A. Kuc, L. Schade, R. D. Johnson, M. Dollmann, P. K. Nayak, H. J. Snaith, P. Radaelli, R. Kudrawiec, R. J. Nicholas, P. Plochocka,

M. Baranowski, Revealing the nature of photoluminescence emission in the metal-halide double perovskite $Cs_2AgBiBr_6$. *J. Mater. Chem. C* **7**, 8350–8356 (2019).

36. A. D. Wright, L. R. V. Buizza, K. J. Savill, G. Longo, H. J. Snaith, M. B. Johnston, L. M. Herz, Ultrafast excited-state localization in $Cs_2AgBiBr_6$ double perovskite. *J. Phys. Chem. Lett.* **12**, 3352–3360 (2021).

37. A. Létoublon, S. Paofai, B. Rufflé, P. Bourges, B. Hehlen, T. Michel, C. Ecolivet, O. Durand, S. Cordier, C. Katan, J. Even, Elastic constants, optical phonons, and molecular relaxations in the high temperature plastic phase of the $CH_3NH_3PbBr_3$ hybrid perovskite. *J. Phys. Chem. Lett.* **7**, 3776–3784 (2016).

38. C. Paillard, S. Prosandeev, L. Bellaiche, Ab initio approach to photostriction in classical ferroelectric materials. *Phys. Rev. B* **96**, 045205 (2017).

39. C. Paillard, L. Bellaiche, Light: A new handle to control the structure of cesium lead iodide. *Phys. Rev. B* **107**, 054107 (2023).

40. W. Zhong, D. Vanderbilt, Competing structural instabilities in cubic perovskites. *Phys. Rev. Lett.* **74**, 2587–2590 (1995).

41. S. A. Hayward, E. K. H. Salje, Cubic-tetragonal phase transition in $SrTiO_3$ revisited: Landau theory and transition mechanism. *Phase Transit.* **68**, 501–522 (1999).

42. N. A. Pertsev, A. K. Tagantsev, N. Setter, Phase transitions and strain-induced ferroelectricity in $SrTiO_3$ epitaxial thin films. *Phys. Rev. B* **61**, R825–R829 (2000).

43. R. He, H. Wu, L. Zhang, X. Wang, F. Fu, S. Liu, Z. Zhong, Structural phase transitions in $SrTiO_3$ from deep potential molecular dynamics. *Phys. Rev. B* **105**, 064104 (2022).

44. X. Ouyang, W. Chen, Y. Zhang, F. Zhang, Y. Zhuang, X. Jie, L. Liu, D. Wang, Structural phase transition involving octahedron tilting and ion migration in metal-halide perovskites: A machine-learning study. *Phys. Rev. B* **108**, L020103 (2023).

45. S. Jung, T. Birol, Structural phase transitions in $SrTiO_3$ from deep potential molecular dynamics. *Nano Lett.* **25**, 3240–3246 (2025).

46. D. Xinyuan, J. Li, G. Niu, J.-H. Yuan, K.-H. Xue, M. Xia, W. Pan, X. Yang, B. Zhu, J. Tang, Lead halide perovskite for efficient optoacoustic conversion and application toward high-resolution ultrasound imaging. *Nat. Commun.* **12**, 3348 (2021).

47. A. H. Slavney, T. Hu, A. M. Lindenberg, H. I. Karunadasa, A bismuth-halide double perovskite with long carrier recombination lifetime for photovoltaic applications. *J. Amer. Chem. Soc.* **138**, 2138–2141 (2016).

48. M. Armer, J. Hocker, C. Buchner, S. Hafele, P. Dörflinger, M. T. Sirtl, K. Tvingstedt, T. Bein, V. Dyakonov, Influence of crystallisation on the structural and optical properties of lead-free $Cs_2AgBiBr_6$ perovskite crystals. *CrstEngComm* **23**, 6848–6854 (2021).

49. H. J. Jöbsis, V. M. Caselli, S. H. C. Askes, E. C. Garnett, T. J. Savenije, T. Freddy, E. M. Hutter, Recombination and localization: Unfolding the pathways behind conductivity losses in $Cs_2AgBiBr_6$ thin films. *Appl. Phys. Lett.* **119**, 131908 (2021).

50. Y. Lun, J. Liu, B. Wei, Z. Gao, X. Wang, J. Hong, Elastic properties of photovoltaic single crystal $Cs_2AgBiBr_6$. *Exp. Mech.* **62**, 117–123 (2022).

51. V. Gusev, A. Karabutov, *Laser Optoacoustics* (American Institute of Physics, 1993).

52. Y. Yan, J. E. B. Gamble, K. A. Nelson, Impulsive stimulated scattering: General importance in femtosecond laser pulse interactions with matter, and spectroscopic applications. *J. Chem. Phys.* **83**, 5391–5399 (1985).

53. V. Gusev, P. Picart, D. Mounier, J.-M. Breteau, On the possibility of ultrashort shear acoustic pulses excitation due to the laser-induced electrostrictive effect. *Opt. Commun.* **204**, 229–236 (2002).

54. O. Matsuda, T. Tachizaki, T. Fukui, J. J. Baumberg, O. B. Wright, Acoustic phonon generation and detection in $GaAs/Al_{0.3}Ga_{0.7}As$ quantum wells with picosecond laser pulses. *Phys. Rev. B* **71**, 115330 (2005).

55. Y. Zhou, L. You, S. Wang, Z. Ku, H. Fan, D. Schmidt, A. Rusydi, L. Chang, L. Wang, P. Ren, L. Chen, G. Yuan, L. Chen, J. Wang, Giant photostriction in organic–inorganic lead halide perovskites. *Nat. Commun.* **7**, 11193 (2016).

56. A. Authier, *International Tables for Crystallography, Volume D: Physical Properties of Crystals* (Kluwer, ed. 1, 2003).

57. T. J. Jacobsson, L. J. Schwan, M. Ottosson, A. Hagfeldt, T. Edvinsson, Determination of thermal expansion coefficients and locating the temperature-induced phase transition in methylammonium lead perovskites using X-ray diffraction. *Inorg. Chem.* **54**, 10678–10685 (2015).

58. A. E. J. Hoffman, R. A. Saha, S. Borgmans, P. Puech, T. Braeckevelt, M. B. J. Roeffaers, J. A. Steele, J. Hofkens, V. van Speybroeck, Understanding the phase transition mechanism in the lead halide perovskite $CsPbBr_3$ via theoretical and experimental GIWAXS and Raman spectroscopy. *APL Materials* **11**, 041124 (2023).

59. K. R. Subbaswamy, A. A. Maradudin, Photoelastic and surface-corrugation contributions to Brillouin scattering from an opaque crystal. *Phys. Rev. B* **18**, 4181–4199 (1978).

60. D. F. Nelson, M. Lax, New symmetry for acousto-optic scattering. *Phys. Rev. Lett.* **24**, 379–380 (1970).

61. E. Anastassakis, E. Burstein, The full symmetry of the photoelastic tensor of elastic waves. *J. Phys. C. Solid Stat. Phys.* **7**, 1374–1380 (1974).

62. R. Vacher, L. Boyer, Brillouin scattering: A tool for the measurement of elastic and photoelastic constants. *Phys. Rev. B* **6**, 639–673 (1972).

63. M. Jäckl, V. I. Belotelov, I. A. Akimov, I. V. Savochkin, D. R. Yakovlev, A. K. Zvezdin, M. Bayer, Magnon accumulation by clocked laser excitation as source of long-range spin waves in transparent magnetic films. *Phys. Rev. X* **7**, 021009 (2017).

64. M. Kobecki, A. V. Scherbakov, T. L. Linnik, S. M. Kukhtaruk, V. E. Gusev, D. P. Pattnaik, I. A. Akimov, A. W. Rushforth, A. V. Akimov, M. Bayer, Resonant thermal energy transfer to magnons in a ferromagnetic nanolayer. *Nat. Commun.* **11**, 4130 (2020).

65. M. N. Tran, R. S. Rodriguez, J. R. Geniesse, K. Sandrakumar, I. J. Cleveland, E. S. Aydil, Stability of $Cs_2NaBiBr_6$ and $Cs_2NaBiCl_6$. *Inorg. Chem.* **63**, 12818–12825 (2024).

66. F. Pelle, B. Blanzat, B. Chevalier, Low temperature phase transition in cubic elpasolithe crystal $Cs_2NaBiCl_6$. *Solid State Commun.* **49**, 1089–1093 (1984).

67. S. Banerjee, S. Saikia, M. S. Molokeev, A. Nag, Unveiling temperature-induced structural phase transition and luminescence in Mn^{2+}-Doped $Cs_2NaBiCl_6$ double perovskite. *Chem. Mater.* **36**, 4750–4757 (2024).

68. A. Noculak, V. Morad, K. M. McCall, S. Yakunin, Y. Shynkarenko, M. Wörle, M. V. Kovalenko, Bright blue and green luminescence of Sb(III) in double perovskite Cs_2MInCl_6 (M = Na, K) matrices. *Chem. Mater.* **32**, 5118–5124 (2020).

69. Y. Liu, Y. Mao, Z. Zhang, K. Liu, D. Xu, J. Zhou, Doping lanthanides towards improvement in self-trapped excitons emission and multiple emission color of Cs_2KInCl_6 double perovskites. *Ceram. Int.* **51**, 16749–16757 (2025).

70. S. Poncé, M. Schlipf, F. Giustino, Origin of low carrier mobilities in halide perovskites. *ACS Energy Lett.* **4**, 456–463 (2019).

71. A. Cohen, T. M. Brenner, J. Klarbring, R. Sharma, D. H. Fabini, R. Korobko, P. K. Nayak, O. Hellman, O. Yaffe, Diverging expressions of anharmonicity in halide perovskites. *Adv. Mater.* **2**, 2463–2469 (2017).

72. A. H. Slavney, L. Leppert, D. Bartesaghi, A. Gold-Parker, M. F. Toney, T. J. Savenije, J. B. Neaton, H. I. Karunadasa, Defect-induced band-edge reconstruction of a bismuth-halide double perovskite for visible-light absorption. *J. Amer. Chem. Soc.* **139**, 5015–5018 (2017).

73. P. Blaha, K. Schwarz, F. Tran, R. Laskowski, G. K. H. Madsen, L. D. Marks, WIEN2k: An APW+lo program for calculating the properties of solids. *J. Chem. Phys.* **152**, 074101 (2020).

74. M. Jamal, M. Bilal, I. Ahmad, S. Jalali-Asadabadi, IRelast package. *J. Alloy. Compd.* **735**, 569–579 (2018).

75. M. Simenas, A. Gagor, J. Banys, M. Maczka, Phase transitions and dynamics in mixed three- and low-dimensional lead halide perovskites. *Chem. Rev.* **124**, 2281–2326 (2024).

76. M. Kulbak, D. Cahen, G. Hodes, How important is the organic part of lead halide perovskite photovoltaic cells efficient $CsPbBr_3$ cells. *J. Phys. Chem. Lett.* **6**, 2452–2456 (2015).

77. F. Pan, J. Li, X. Ma, Y. Nie, B. Liu, H. Ye, Free and self-trapped exciton emission in perovskite $CsPbBr_3$ microcrystals. *RSC Adv.* **12**, 1035–1042 (2021).

78. J. A. Peters, Z. Liu, M. C. De Siena, M. G. Kanatzidis, B. W. Wessels, Photoluminescence spectroscopy of excitonic emission in $CsPbCl_3$ perovskite single crystals. *J. Lumin.* **243**, 118661 (2022).

79. Y. Su, X. Chen, W. Ji, Q. Zeng, Z. Ren, Z. Su, L. Liu, Highly controllable and efficient synthesis of mixed-halide $CsPbX_3$ (X = Cl, Br, I) perovskite qds toward the tunability of entire visible light. *ACS Appl. Mater. Interfaces* **9**, 33020–33028 (2017).

80. A. Anselm, *Introduction to Semiconductor Theory* (Prentice Hall, 1982).
